# Shared retinoic acid responsive enhancers coordinately regulate nascent transcription of *Hoxb* coding and non-coding RNAs in the developing mouse neural tube

**DOI:** 10.1242/dev.201259

**Published:** 2023-05-24

**Authors:** Zainab Afzal, Jeffrey J. Lange, Christof Nolte, Sean McKinney, Christopher Wood, Ariel Paulson, Bony De Kumar, Jay Unruh, Brian D. Slaughter, Robb Krumlauf

**Affiliations:** ^1^ Stowers Institute for Medical Research, Kansas City, MO 64110, USA; ^2^ Anatomy and Cell Biology Department, Kansas University Medical Center, Kansas City, KS 66160, USA

**Keywords:** Hox genes, Transcriptional regulation, Shared enhancers, Nascent transcription, smFISH, Retinoic acid signaling, Retinoic acid response elements, RAREs, Neural tube, Mouse development

## Abstract

Signaling pathways regulate the patterns of Hox gene expression that underlie their functions in the specification of axial identity. Little is known about the properties of *cis*-regulatory elements and underlying transcriptional mechanisms that integrate graded signaling inputs to coordinately control Hox expression. Here, we optimized a single molecule fluorescent *in situ* hybridization (smFISH) technique with probes spanning introns to evaluate how three shared retinoic acid response element (RARE)-dependent enhancers in the *Hoxb* cluster regulate patterns of nascent transcription *in vivo* at the level of single cells in wild-type and mutant embryos. We predominately detect nascent transcription of only a single *Hoxb* gene in each cell, with no evidence for simultaneous co-transcriptional coupling of all or specific subsets of genes. Single and/or compound RARE mutations indicate that each enhancer differentially impacts global and local patterns of nascent transcription, suggesting that selectivity and competitive interactions between these enhancers is important to robustly maintain the proper levels and patterns of nascent *Hoxb* transcription. This implies that rapid and dynamic regulatory interactions potentiate transcription of genes through combined inputs from these enhancers in coordinating the retinoic acid response.

## Teaser

**Highlighted Article:** Rapid and dynamic enhancer-promoter interactions in the *Hoxb* cluster potentiate transcription of individual genes through combined inputs from shared enhancers in coordinating the response to retinoic acid.

## INTRODUCTION

Hox genes encode a conserved family of transcription factors (TFs) that play important roles in regulating regional identity of tissues along the anterior-posterior (A-P) axis (Lewis, 1978; Duboule and Dollé, 1989; Graham et al., 1989; McGinnis and Krumlauf, 1992; Carroll, 1995; Mallo et al., 2010; Martin et al., 2016; Arendt, 2018; He et al., 2018). Hox genes are arranged in clusters and conserved features in their organization result in the generation of highly ordered patterns of Hox expression and function along the A-P axis (Lewis, 1978; Duboule and Dollé, 1989; Graham et al., 1989; Serano et al., 2016). Through coordinate regulation of their spatial and temporal patterns of expression, Hox genes lay down a combinatorial code that is important in the regulatory hierarchy that specifies the regional properties of tissues and modulates morphological diversity. Disruptions in the expression domains of Hox genes result in homeotic transformations and altered morphogenesis (Balkaschina, 1929; Bridges and Dobzhan, 1933; Garber et al., 1983; Hafen et al., 1984; Pultz et al., 1988; Merrill et al., 1989; Maconochie et al., 1996; Philippidou and Dasen, 2013; Quinonez and Innis, 2014). Therefore, the levels and patterns of Hox gene expression along the A-P axis must be precisely regulated in space and time for proper elaboration of the basic body plan. In embryogenesis, graded cues from signaling pathways, such as retinoic acid (RA), Wnt (wingless related integration site) and Fgf (fibroblast growth factors), play a key role in organizing the patterns of Hox gene expression that underlie their functions in specification of axial identity and assignment of cell fate (Simeone et al., 1990; Pownall et al., 1996; Bel-Vialar et al., 2002; Diez del Corral and Storey, 2004; Deschamps and van Nes, 2005; Schilling et al., 2012; Neijts et al., 2016; Deschamps and Duboule, 2017; Darras et al., 2018; Frank and Sela-Donenfeld, 2019; Nolte et al., 2019). Therefore, it is important to understand the properties of *cis*-regulatory elements and transcriptional mechanisms that interpret these graded signaling inputs to coordinately control the dynamics of Hox expression.

Regulatory analyses in invertebrate and vertebrate model systems have revealed that diverse mechanisms provide inputs that control patterns of Hox expression at the level of transcription (de Laat and Duboule, 2013; Gregor et al., 2014; Gentile and Kmita, 2018; Afzal and Krumlauf, 2022; Batut et al., 2022; Gaskill and Harrison, 2022). In vertebrates, regulation has been linked to a variety of *cis*-regulatory elements (CREs) and differences in chromatin states, epigenetic modifications and chromosome topology (Sharpe et al., 1998; Chambeyron and Bickmore, 2004; Tarchini and Duboule, 2006; Gonzalez et al., 2007; Soshnikova and Duboule, 2009; Noordermeer et al., 2011, 2014; Mazzoni et al., 2013; Ahn et al., 2014; De Kumar et al., 2015; Narendra et al., 2015, 2016; Neijts et al., 2016; Parker and Krumlauf, 2017; Qian et al., 2018; Rodríguez-Carballo et al., 2019). Among the CREs, enhancers play an important role in modulating the activation and/or maintenance of Hox gene transcription through their ability to interpret graded cues from signaling pathways and to integrate combinations of TFs that control gene expression patterns in a spatio-temporal and tissue-specific manner (Marshall et al., 1994; Studer et al., 1994; Tümpel et al., 2002; Berlivet et al., 2013; Delpretti et al., 2013; Paris et al., 2013; Crocker et al., 2015; Heinz et al., 2015; Parker and Krumlauf, 2017; Henriques et al., 2018; Nolte et al., 2019; Parker et al., 2019; Choi et al., 2021; Kreibich et al., 2022). Studies in mice have demonstrated that there are multiple enhancers embedded within and flanking the Hox clusters that can exhibit overlapping activities (shadow enhancers), selective and competitive preferences for target genes, and regulate both near adjacent genes or act more globally on multiple genes in a cluster (shared enhancers) (Oosterveen et al., 2003a; Scotti and Kmita, 2012; Tschopp et al., 2012; Andrey et al., 2013; Berlivet et al., 2013; Nolte et al., 2013; Ahn et al., 2014; Qian et al., 2018). The different roles of enhancers in regulation of Hox genes, in conjunction with the compact nature and relatively high density of transcriptional units in Hox clusters, raises fundamental questions about the specificity of enhancers and how they locate and distinguish between their target loci (Sanyal et al., 2012; Long et al., 2016; Furlong and Levine, 2018; Schoenfelder and Fraser, 2019; Zeitlinger, 2020; Afzal and Krumlauf, 2022; Batut et al., 2022; Gaskill and Harrison, 2022; Levo et al., 2022).

Models such as linking (Morcillo et al., 1997), tracking (Kong et al., 1997) and looping (Dunn et al., 1984; Deng et al., 2012) have been proposed to explain how enhancers can locate and activate gene promoters. It is unclear whether there are optimal enhancer-promoter distances and if such preferences are linked to regulation of spatio-temporal or tissue-specific activities in development (Bartman et al., 2016; Benabdallah et al., 2019). Recent studies have provided evidence for rapid dynamics in interactions between enhancers and their target promoters over a wide range of distances, which has led to a revision of models postulating stable long-term enhancer-promoter interactions in favor of models based on dynamic looping (Bothma et al., 2014; Gregor et al., 2014; Furlong and Levine, 2018; Liu and Tjian, 2018; Mir et al., 2018; Zhang and Tjian, 2018; Berrocal et al., 2020; Eck et al., 2020; Zuin et al., 2022). There is evidence for the occurrence of dynamic co-transcriptional hubs containing shared pools of components of the general transcription machinery and upstream activators (Tsai et al., 2017, 2019; Furlong and Levine, 2018; Mir et al., 2018). In *Drosophila*, many shared enhancers of developmental genes have recently been shown to co-transcriptionally couple activity of their target genes in living embryos, even if those genes are separated by large distances, by integrating independent inputs from tethering elements and topologically associated domains (TADs) (Levo et al., 2022). This mode of genome organization creates what are referred to as ‘topological operons’ for coordinate and co-dependent transcriptional regulation of multiple genes by shared enhancers. Whether shared enhancers in mammals and other vertebrates also tend to regulate their target genes by similar co-transcriptional coupling mechanisms is unknown.

**Fig. 1.**
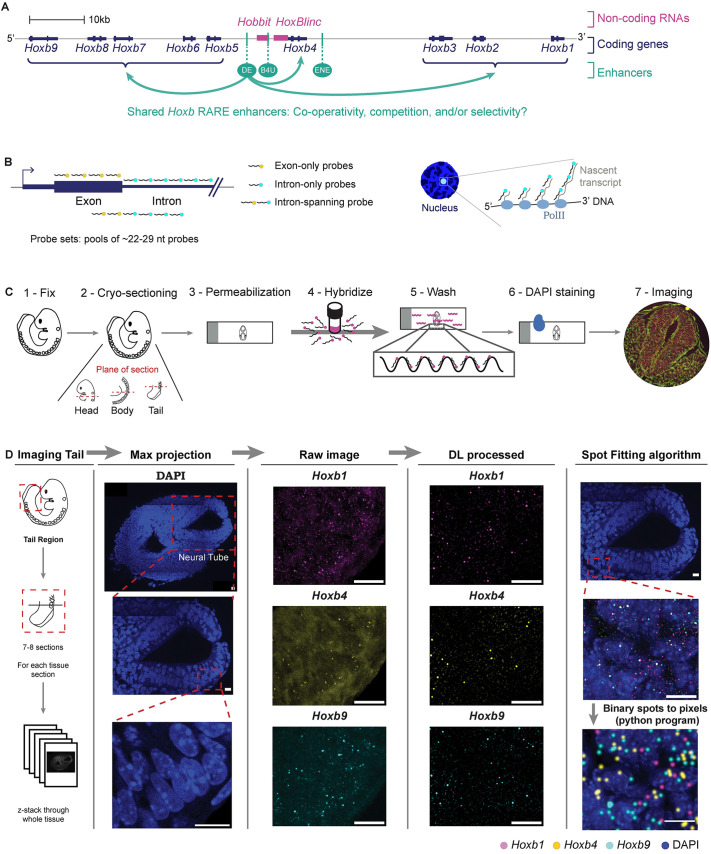
**Optimized smFISH pipeline for detecting and processing nascent transcripts at the level of single cells in mouse embryonic tissues.** (A) Schematic of the *Hoxb* cluster, drawn to scale. Previous evidence has shown that *DE* (arrows drawn) and *ENE* are able to globally regulate the steady state levels of genes in the cluster. (B) Probes designed against gene introns and exons to detect nascent transcripts. (C) Schematic for optimized single molecule fluorescent *in situ* hybridization (smFISH) technique, to detect nascent transcripts in cryo-sectioned mouse embryos. (D) Deep Learning (DL) pipeline to quantify nascent transcripts. A region of the neural tube is magnified to show the raw image and the DL processed image. The DL processed image can be spot fitted to increase the size of nascent spots for better visualization. Scale bars: 10 μm.

The mouse *Hoxb* cluster provides a good context for investigating the properties and roles of shared enhancers in transcriptional regulation of Hox genes in response to RA signaling. The genes are located within a single TAD, encompassing many sub-TADs (Dixon et al., 2012; Ke et al., 2017). Dynamic RA gradients directly activate *Hoxb* genes in the developing hindbrain, spinal cord, hematopoietic stem cells (HSCs) and other tissues (Bel-Vialar et al., 2002; Oosterveen et al., 2003a; Deschamps and van Nes, 2005; Sirbu et al., 2005; Niederreither and Dollé, 2008; Bertrand et al., 2011; Rhinn and Dollé, 2012; Zaffran and Kelly, 2012; Nolte et al., 2013, 2019; Deschamps and Duboule, 2017; Qian et al., 2018; Krumlauf and Wilkinson, 2021) and the transcriptional responses are interpreted in part through a series of retinoic acid response elements (RAREs) embedded within and flanking the cluster (Marshall et al., 1994; Studer et al., 1994; Dupé et al., 1997; Gould et al., 1998; Houle et al., 2003; Nolte et al., 2003, 2013, 2019; Oosterveen et al., 2003b; Ahn et al., 2014). For example, conserved RAREs (*DE*, *B4U* and *ENE*) are components of three enhancers present in a 15 kb region in the middle of the cluster, which also contains transcription units for *Hoxb4*, *Hoxb5*, *mir10a* and two long non-coding (lnc) RNAs implicated in regulation of *Hoxb* genes (*Hobbit* and *HoxBlinc*) (De Kumar et al., 2015; De Kumar and Krumlauf, 2016; Deng et al., 2016; Degani et al., 2021) (Fig. 1A). These three shared enhancers coordinate global *Hoxb* responses to RA by regulating multiple genes (Gould et al., 1997, 1998; Sharpe et al., 1998; Oosterveen et al., 2003a,b; Ahn et al., 2014). The *DE*-RARE is subject to epigenetic modifications, and is required for coordinating global regulation of the *Hoxb* complex in HSCs and the developing neural tube, as indicated by arrows in Fig. 1A (Ahn et al., 2014; De Kumar et al., 2015; Qian et al., 2018). The *ENE*-RARE regulates *Hoxb4* and *Hoxb3* and transgenic analyses suggest some degree of functional overlap with the *DE*-RARE in regulating other genes in the cluster (Gould et al., 1997; Ahn et al., 2014). This high density of enhancers and transcription units in such a small region (Fig. 1A) raises questions regarding target specificity and selective preferences of the shared enhancers and whether coordinate regulation occurs through co-transcriptional coupling of *Hoxb* genes. Whether these three enhancers exert their effects in an independent manner or though coordinated interactions with each other is not clear.

To begin to address these questions, we analyzed patterns of *de novo* (nascent) transcription of multiple *Hoxb* transcription units at the level of individual cells in mouse embryos. We employed a single molecule florescent *in situ* hybridization (smFISH) technique (Raj and Tyagi, 2010) in conjunction with probes spanning introns, and optimized it for use in tissue sections from stage matched wild-type and mutant mouse embryos to assess the *in vivo* transcriptional activity state of genes within the cluster. We developed a deep learning (DL) algorithm for automatic calling and localization of signals for nascent transcripts in the nucleus which allowed us to systematically quantify and compare patterns of nascent transcripts within the *Hoxb* cluster and their co-occurrence in individual cells. We see no evidence for co-transcriptional coupling of all genes in the cluster in an individual cell and predominately detect nascent transcription of a single gene, with a much lower frequency of simultaneous transcription of one or two other transcriptional units. We also generated mouse lines carrying a series of single and compound mutations in the *DE*, *B4U* and *ENE* RAREs and found that each of the three enhancers differentially impacts patterns of nascent transcription of *Hoxb* transcripts. Two enhancers have a more global impact on transcription, whereas the third plays a predominantly local role impacting transcription patterns in the cluster. Our results suggest that selectivity and competitive interactions between enhancers plays a role in coordinating patterns of nascent transcription of Hox genes in the neural tube and provides new insights into the properties of shared enhancers in coordinating the *Hoxb* transcriptional dynamics during development.

## RESULTS

### Developing an *in vivo* approach to study transcriptional regulation of the *Hoxb* cluster by shared enhancers

In this study we investigate the properties of three shared RA-dependent enhancers (*DE*, *B4U* and *ENE*) present in the *Hoxb* cluster (Fig. 1A) and their input in regulating patterns of nascent transcription *in vivo* at the level of single cells in mouse embryos. The high density of enhancers and transcription units in the *Hoxb* cluster raises questions regarding promoter target selection, the degree to which these three enhancers individually or collectively participate in modulating short and long-range regulation of multiple genes and whether there is co-transcriptional coupling of regulated transcriptional units.

To address these questions, we wanted to analyze *in vivo* patterns and sites of active transcription in embryos, which can be visualized by detecting nascent transcripts in individual cells. We employed an smFISH technique (Raj and Tyagi, 2010) and optimized parameters to facilitate its use in fixed tissue sections from stage matched wild-type and mutant mouse embryos (Fig. 1C). To visualize nascent nuclear transcripts, we designed and validated probe sets spanning introns for genes of interest. In some cases, probes only contained intron sequences, while in other cases the probes spanned intron and some exonic sequence, due to constraints of gene size (Fig. 1B; Table S1). This allowed us to monitor and quantify patterns of nascent transcription at a defined stage in specific tissues and assess the *in vivo* transcriptional activity state of both coding and non-coding genes within the cluster at the level of individual cells. To ensure the specificity of probes in detecting nascent nuclear transcripts, we required that the probes for each transcript only showed one or two nuclear spots in a cell and have a high signal-to-background ratio (Fig. 1D).

For comparative analyses, we employed multiplexed probe sets combining probes for up to three different transcripts and used DAPI staining to identify nuclei. Using alternate sections for different probe sets, we monitored and compared nascent transcription patterns of up to six transcription units within a defined region of the same embryo. Imaging through the whole tissue section (10 µm) allowed us to visualize all nascent transcripts present in the section. We developed a DL method to identify and quantify nascent transcripts in a high-throughput and unbiased manner (Fig. 1D), which allowed us to systematically quantify and compare patterns of nascent transcription of *Hoxb* genes and their co-occurrence in individual cells.

The DL output has a confidence probability between 0 and 1, for whether or not each detected fluorescent spot is a nascent transcript, over the whole tissue section. To test network performance, we compared DL detected spots against spots manually and independently marked by several people on the same sections. There was an 80% overlap between different people marking nascent spots, and between manually marked spots compared with those detected by DL on the same sections (Fig. S1A). Hence, the DL network is as reliable as manual detection of nascent spots. Heatmaps for the intensities of nascent transcripts over the whole tissue section were also generated for each probe (Fig. S2A). To better visualize nascent transcripts over the whole tissue, we performed spot fitting on DL-detected nascent transcripts which allowed us to increase the size of the nascent spots and overlay them onto DAPI staining (Fig. S2B).

**Fig. 2.**
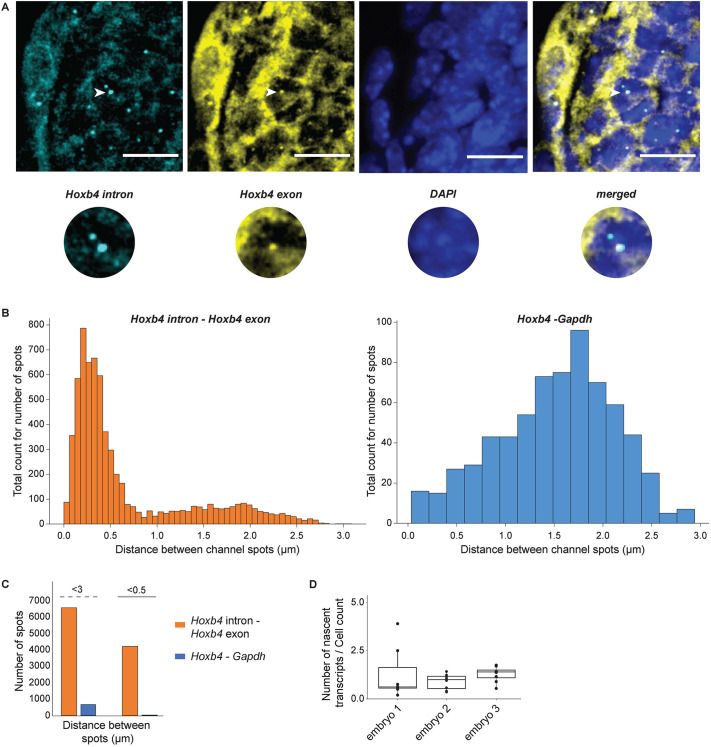
**Validation of smFISH pipeline used to process and quantify nascent transcripts in tissue samples.** (A) Image of *Hoxb4* intron and exon probes. White arrowheads point at nascent transcripts that are also shown in the magnified inset. Scale bars: 10 μm. (B) Plot for closest distance between nascent transcripts from *Hoxb4* intron and exon probes compared against *Hoxb4*-*Gapdh* probes. (C) Histograms drawn for the distance between spots in the two channels: 3 μm for nuclear radius and 0.5 μm for the co-localization of two spots. (D) Average *Hoxb4* nascent spots in tail sections of multiple embryos. Box plot shows the median as the central line, the first and third quartiles as the box, and the upper and lower whiskers extend from the quartile box to the largest/smallest value within 1.5× of the interquartile range; dots indicate outlier tissue sections.

To validate the approach, we hybridized the same tissue section with a probe set for the *Hoxb4* intron, which would only detect nascent transcripts, and one spanning the exon and intron, which would detect both nascent and mature transcripts, and observed a high degree of overlap in nascent signals with both probe sets (Fig. 2A, magnified insets). The *Hoxb4* intron probe showed brighter localized nuclear fluorescent spots with minimal background, whereas the exon probe detected both nascent transcripts in the nucleus and weaker signals in the cytoplasm. The cytoplasmic signal appears to represent mature RNA transcripts but due to background autofluorescence present in the tissue section we were unable to robustly differentiate and count these signals. Hence, a caveat of our optimized smFISH protocol is that we could not quantify single mature transcripts or measure total fluorescent intensity to infer transcriptional rates for genes (Fig. S1B). Therefore, in all smFISH analyses we focused on visualizing and quantifying only patterns of nascent transcription.

To further quantify the accuracy and robustness of our approach along with specificity of probes, we calculated the overlap of different *Hoxb4* intron and exon probe sets (Fig. 2A). We compared distances between spots observed from *Hoxb4* intron versus exon probes against the distance between spots observed for the *Hoxb4-Gapdh* control probes. In tissue sections, we cannot demarcate cell and nuclear boundaries. Therefore in each section we identified all high confidence spots in the specific channel for each individual probe set and fitted these spots to 2D Gaussians to find their exact centers. Then we searched in a 3.9×3.9 µm square around each of these spots for the presence of spots in channels from the other probes and calculated the distance between the centers of these high confidence signals (Fig. 2B). As expected for probes against the same transcript, we saw that the majority of co-localized spots detected by *Hoxb4* intron and exon probes were less than 500 nm apart, and the average distance between spots was 319 nm. In contrast, the distance between spots of nascent transcription for the *Hoxb4* and *Gapdh* genes was over 1.5 µm (Fig. 2B). We quantified the fraction of spots that were co-localized less than 500 nm apart and found that 64.5% of all *Hoxb4* exon spots co-localized with a spot detected by the intron probe, whereas only 6.75% of *Hoxb4* spots had a *Gapdh* spot less than 500 nm away (Fig. 2C).

To facilitate reproducibility, all 9.5 days post coitum (dpc) mouse embryos in smFISH analyses were at the same specific stage based on their having an identical number of somites (24 somites). To examine consistency between embryos we compared the numbers of nascent *Hoxb4* transcripts in the tail sections of the neural tube from three different embryos (Fig. 2D). Although there are some small differences in numbers of nascent transcripts observed, the averages are not significantly different between the three sets of sections. Therefore, we can robustly quantify expression of multiple nascent transcripts and use averages of sections in a defined axial region for comparisons across different embryos.

### Patterns of nascent *Hoxb* transcription in wild-type embryos

**Fig. 3.**
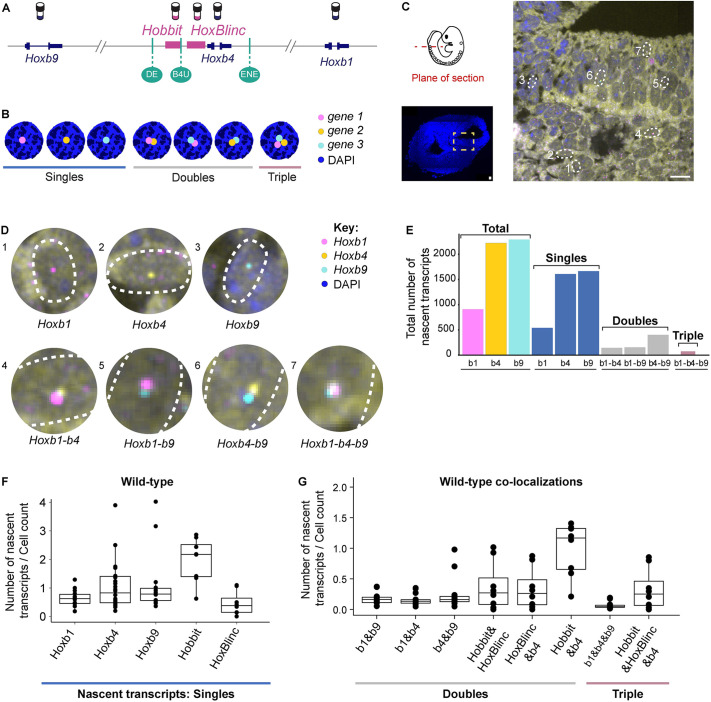
**Quantifying patterns of nascent transcription of coding and non-coding genes from the *Hoxb* complex in wild-type embryos.** (A) smFISH probes designed against the ends and center of the *Hoxb* cluster. (B) Schematic for possible combinations of nascent transcripts that can be observed when hybridizing with three genes simultaneously. (C) Cross-section through one tissue sample from the tail region of a 9.5 dpc mouse embryo. Magnified region shows nascent transcripts present in portion of the neural tube. Scale bars: 10 μm. (D) Amplified cell insets from the image in C with spots marking possible combinations of nascent transcripts of *Hoxb* coding genes. (E) Bar plot comparing the total number of nascent *Hoxb* transcripts measured for each gene in the single tail section shown in C and D. (F) Box plot comparing the total number of nascent transcripts/cell for *Hoxb* transcripts calculated as an average of data from multiple near adjacent tail sections (7-9) of the same embryo. (G) Box plot showing the total number of co-localized double and triple combinations of nascent transcripts/cell for *Hoxb* genes calculated as an average of data from multiple near adjacent tail sections (7-9) of the same embryo. Box plots show the median as the central line, the first and third quartiles as the box, and the upper and lower whiskers extend from the quartile box to the largest/smallest value within 1.5× of the interquartile range; dots indicate outlier tissue sections.

We applied this approach to compare patterns of nascent transcription of multiple transcription units in the *Hoxb* cluster by designing and validating probe sets for other coding and non-coding RNAs (Fig. 3A; Table S1). In addition to *Hoxb4*, we generated robust probe sets for detecting nascent transcripts of the *Hoxb1* and *Hoxb9* genes at either end of the cluster, and for the lncRNAs, *Hobbit* and *HoxBlinc*, present in the center of the cluster. We attempted to generate probes for several other *Hoxb* genes, but encountered issues with high backgrounds, specificity and reproducibility. Therefore, we focused our analyses using the robust and specific probes sets we developed.

Two different probe set combinations were used on a series of alternating tissue sections to examine the patterns of nascent transcription in directly comparable regions across the whole embryo. Probe set 1 consisted of *Hoxb1*, *Hoxb4* and *Hoxb9*, and probe set 2 consisted of *Hobbit*, *HoxBlinc* and *Hoxb4*. *Hoxb4* served as an internal control in both probe sets to verify comparable expression patterns when quantifying nascent transcripts across alternate sections (Fig. S3A). This allowed quantification for five different transcription units in the posterior neural tube of each mouse embryo. There was some minor variation between biological samples of embryos and in expression along the A-P axis, but the patterns compared very well when analyses were conducted at the same axial level across samples (Fig. S3B). As another means of evaluating this method, we quantified the patterns of nascent transcription of the three *Hoxb* coding genes along the A-P axis in wild-type embryos by generating sections from the head, trunk and tail regions (Fig. S4) and compared them with known profiles of their steady state expression determined by conventional colorimetric *in situ* analyses (Goh et al., 1997; Maconochie et al., 1997; Gould et al., 1998; Folberg et al., 1999; Brend et al., 2003; Medina-Martínez and Ramírez-Solis, 2003). Results were in good agreement with the expected spatial order of expression along the A-P axis.

We performed analyses on the neural tube in the tail region where all the *Hoxb* coding genes and lncRNAs are known to be expressed. In each of the two multiplexed probe sets we simultaneously imaged up to three different transcripts. By imaging *z*-slices through a tail section, we detected distinct single spots, where only one of the three loci was undergoing nascent transcription in a cell, and combinations of co-localized spots of nascent transcripts for these genes, which we refer to as singles, doubles and triples (Fig. 3B,C). The co-localized spots of nascent transcription for different combinations of *Hoxb* genes (doubles and triples) in an individual cell represent the occurrence of overlapping transcriptional bursting activities at these loci. Magnified insets illustrate examples of all of the combinations of overlapping nascent transcription observed in the tail section (Fig. 3D). The spots detected by the DL pipeline were quantified and plotted for each section, and the total number of signals for each of the nascent transcripts sub-divided into the categories of singles, doubles or triples, based on their patterns of co-localization (Fig. 3E). For each probe set, ∼7-9 tissue sections from a tail region were imaged, and an estimate of total number of cells present in each neural tube was calculated for each section (see Materials and Methods). The number of nascent transcripts were divided by the cell count, to obtain the average number of spots per cell for all five transcription units identified by the probe sets (Fig. 3F). We also determined the average number of co-localized spots of nascent transcription per cell for all of the double and triple categories (Fig. 3G).

Analyses of the patterns of *de novo* transcriptional bursting activity from a single tail section (Fig. 3E) or from the average of multiple sections (Fig. 3G) clearly show that we predominately detected nascent transcription of a single gene in each cell, with a much lower frequency of simultaneous transcription of one or two other transcriptional units. For example, 72.2% of all sites of nascent transcription detected for *Hoxb4* by the probe sets were found to occur as isolated single spots in a cell, with no evidence for nascent activity of the other *Hoxb* loci (Fig. 3E). In contrast, only 6.5% of *Hoxb4* nascent spots were co-localized with *Hoxb1*, 18.1% with *Hoxb9* and 3.2% with transcriptional bursts of both *Hoxb1* and *Hoxb9*.

It was possible that the number of co-localized spots of nascent transcription we detected in individual cells was simply an outcome of random chance and the activities of each gene were independent of each other. Therefore, we calculated the probability of random chance for co-localized transcripts based on the number of single nascent transcripts for each transcription unit. We found the actual observed co-localization trends were lower than what one would expect by random chance, suggesting there may be an active regulatory process modulating the levels of individual bursting activities (Fig. S5; Table S2).

These results in wild-type embryos indicate that robust co-transcriptional coupling of all genes in the *Hoxb* cluster is not the predominant mode of their coordinate regulation in individual cells, suggesting a rapid and dynamic process for potentiating transcription of individual transcription units. The data show that *Hobbit* and *Hoxb4* transcripts have a higher degree of co-localization than *HoxBlinc* and *Hoxb4* (Fig. 3G). The differences in the patterns for these lncRNAs suggest that their transcriptional activation within the cluster is independently potentiated by distinct inputs.

### Assessing the role of shared enhancers using embryos with RARE mutations

**Fig. 4.**
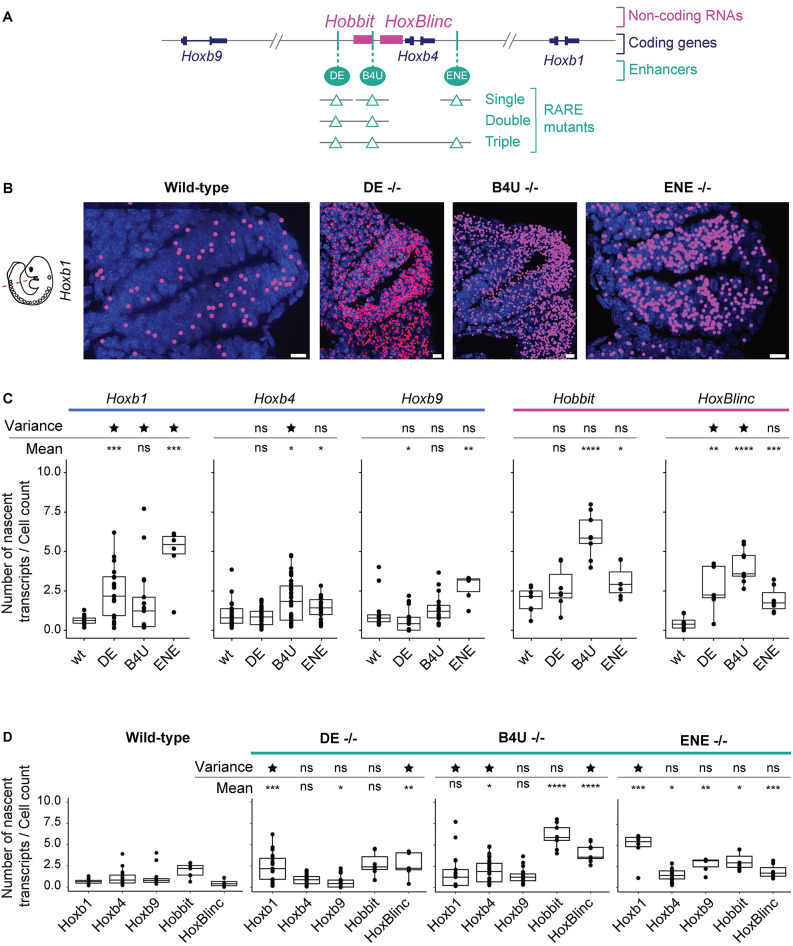
**Quantification of changes in levels and patterns of nascent transcription of *Hoxb* coding and non-coding genes in single mutants of three different shared RARE enhancers.** (A) Diagram depicting a series of single and compound mutants generated in RAREs of three shared enhancers present in the center of the *Hoxb* cluster. (B) Images of nascent transcripts for *Hoxb1* in wild-type and the series of single RARE mutant embryos. The nascent transcripts detected by DL were spot fitted to increase the size of the spots for better visualization. Scale bars: 10 μm. (C) Box plots showing changes in average levels of nascent expression of transcripts measured with two different probe sets on a series of alternate tissue sections from multiple embryos of wild-type and mutant mouse lines. The plots are grouped according to results for each gene. (D) Box plots of data from the same samples as in C, but grouped according to results for each genetic background. Significance is calculated based on both the mean (to estimate how the average expression is changing) as well as for the variance (to estimate how the range of expression among sections is changing) compared with wild-type embryo sections. *P<0.05, **P<0.01, ***P<0.001, ****P<0.0001 (Mann–Whitney test). Star indicates variance *P*-value<0.05 (parametric F-test). ns, not significant (*P*>0.05). Box plots show the median as the central line, the first and third quartiles as the box, and the upper and lower whiskers extend from the quartile box to the largest/smallest value within 1.5× of the interquartile range; dots indicate outlier tissue sections.

We assessed the relative functional contributions of the three shared RA-dependent enhancers (*DE*, *B4U* and *ENE*) on *Hoxb* transcription *in vivo* by generating mouse lines carrying a series of single and compound mutations in their RAREs. Using CRISPR/cas9 gene editing approaches, a series of specific mutations in the *DE-*, *B4U-* and *ENE-*RAREs were created by making base pair substitutions known to disrupt RAR/RXR binding sites (Sucov et al., 1990; Umesono et al., 1991; Oosterveen et al., 2003b; Ahn et al., 2014; Qian et al., 2018), but maintaining the same spatial distances between these and other *cis*-elements in the endogenous locus (Fig. 4A; Table S3). For each single RARE mutant, changes in the average number of nascent transcripts per cell count were visualized in the neural tubes of comparable tissue sections across stage-matched wild-type and RARE mutant embryos. We applied the spot fitting algorithm to aid visualization of the changes occurring in patterns of nascent transcription. Fig. 4B provides an example of the data and changes observed for nascent transcripts of *Hoxb1* in each of the three single RARE mutants compared with wild-type, and results for all five coding and non-coding transcripts are presented in Fig. S6A-D. To facilitate comparisons of the datasets in the different genetic backgrounds, the results were plotted by grouping analyses according to each gene (Fig. 4C) or each individual RARE mutant (Fig. 4D).

This analysis showed that levels of nascent transcription of *Hoxb1*, *Hoxb9* and *HoxBlinc* were altered in *DE*-RARE mutants, with elevated levels for *Hoxb1* and *HoxBlinc*, whereas *Hoxb9* displayed a slight decrease (Fig. 4C,D). In *ENE*-RARE mutants, *Hoxb1*, *Hoxb4*, *Hoxb9* and *Hobbit* all displayed increased levels of nascent transcription. In *B4U*-RARE mutant embryos, levels of *Hoxb4*, *Hobbit* and *HoxBlinc* all displayed an increase over wild-type. These changes imply that all three enhancers have distinct inputs or preferences in regulating *Hoxb* transcripts. The *DE*- and *ENE*-RAREs appear to have a greater long-range role in coordinately regulating transcriptional units spread throughout the cluster, whereas the *B4U*-RARE appears to act more locally in modulating *Hoxb4* and the *Hobbit* and *HoxBlinc* lncRNAs.

The changes in co-regulation of *Hoxb* genes in the mutants are also observed in patterns of co-localization of nascent transcription (Fig. S7). Co-localizations of *Hoxb1*-*Hoxb4* nascent spots are most impacted in the *DE* and *ENE* mutants, whereas *Hoxb1*-*Hoxb9* and *Hoxb4*-*Hoxb9* co-localizations are most impacted in the *ENE* mutant. Hence, mutating the *DE*- and *ENE*-RAREs impacts co-localization patterns of bursting activity of coding genes more than the lncRNAs, and conversely mutation of the *B4U*-RARE changes co-localization patterns for nascent activity of *Hobbit* and *HoxBlinc*, and the coding *Hoxb4* gene.

### Coordinated transcription of *Hoxb* genes is disrupted in compound RARE mutants

It was surprising that the general trend in changes to nascent transcription observed in the single RARE mutants reflected increased levels of transcription of selected subsets of genes rather than decreased activity. The single RARE mutants may change the normal balance of regulatory interactions in the cluster, allowing the remaining enhancers to shift or alter their target preferences, resulting in functional compensation and changes in patterns of transcriptional activation. To investigate the degree to which these enhancers can compensate, antagonize or work together in coordinating transcription, we generated and analyzed compound mutants carrying the same alterations in each RARE.

**Fig. 5.**
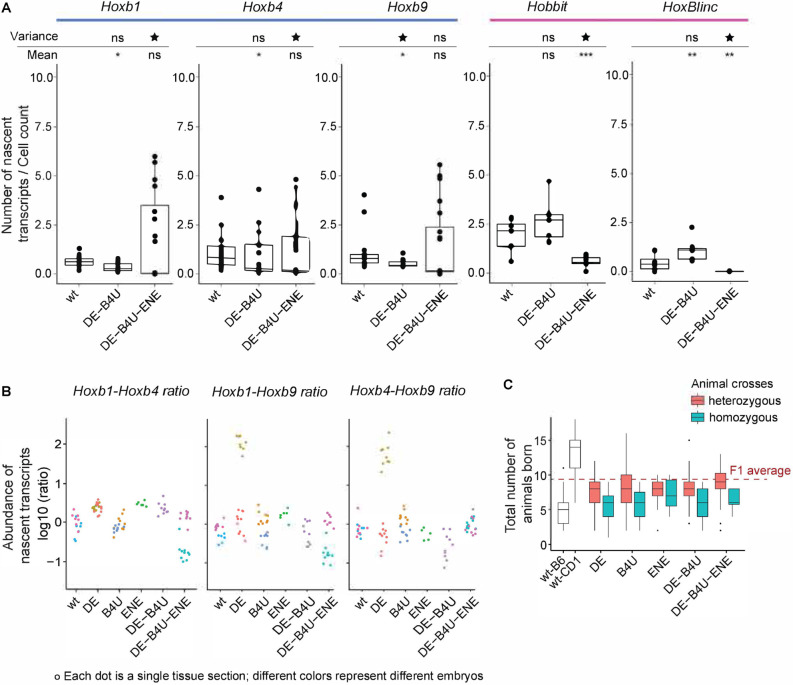
**RARE mutations alter levels and proper coordinated transcription of *Hoxb* genes.** (A) Box plot showing changes in average levels of nascent expression of coding and non-coding *Hoxb* transcripts measured with two different probe sets on a series of alternate tissue sections from multiple embryos of wild-type and compound RARE mutant mouse lines. The plots are grouped according to results for each gene. Significance is calculated based on both the mean as well as for the variance compared with wild-type embryo sections. *P<0.05, **P<0.01, ***P<0.001 (Mann–Whitney test). Star indicates variance *P*-value<0.05 (parametric F-test). ns, not significant (*P*>0.05). (B) Jitter plot for abundance ratios of nascent transcripts/cell plotted for the *Hoxb1*, *Hoxb4* and *Hoxb9* genes. Each dot represents the ratio of counts from the neural tube for one gene over the counts for the second gene in the same section. All sections are for the tail region, and each color is a different embryo. (C) Litter sizes of the single and compound mutants. The two wild-type strains from which F1 strains are generated for injections to make RARE mutants are shown in white. Red dashed line shows approximate litter sizes for the F1 wild-type animals. Box plots show the median as the central line, the first and third quartiles as the box, and the upper and lower whiskers extend from the quartile box to the largest/smallest value within 1.5x of the interquartile range; dots indicate outlier tissue sections.

Analysis of the *DE-B4U* double mutant revealed that the elevated number of nascent transcripts per cell for *Hobbit* and *HoxBlinc* observed in the single RARE mutants unexpectedly returned to near wild-type levels (Figs 4C, 5A). Levels of nascent transcription of *Hoxb1*, *Hoxb4* and *Hoxb9* coding genes were also more similar to that of wild-type embryos. This suggests antagonism or competition between the *DE* and *B4U* enhancers, which is altered in each single mutant, is eliminated in the double mutant. Nascent transcript levels trending towards wild-type in the double mutant implies that the *ENE*-RARE enhancer may functionally compensate for the mutations in the *DE* and *B4U* enhancers to maintain expression, consistent with its global effects on transcription in analysis of single *ENE*-RARE mutants (Fig. 4C,D).

To test this idea and investigate whether all three RAREs are essential, we generated *DE*-*B4U*-*ENE* triple mutant embryos (Fig. 5A). We detected changes and considerable variability in the coordination of nascent transcription of genes in the *Hoxb* cluster, with variable penetrance in the regulatory phenotype. For example, one mutant embryo displayed significantly reduced levels of nascent transcripts for all five genes, below levels in wild-type, consistent with them being required for activity (Fig. 5A). However, in another embryo, the levels and range of expression of nascent transcripts of the *Hoxb* coding genes was equal to or greater than wild-type, but the levels for the *Hobbit* and *HoxBlinc* lncRNAs remained much lower than wild-type. This extensive range of variation in levels of nascent transcripts for coding genes in the triple mutants was generally not observed in our analysis of the single and double mutants. However, we did observe an increase in the range of expression of *Hoxb1* and *HoxBlinc* in single *DE* and *B4U* mutants (Fig. 4C,D). To quantify changes in the range of levels of nascent transcription in the RARE mutants, we calculated whether the variance was significantly different in the series of mutants analyzed compared with wild-type (Fig. S8). The significant changes in variance we detected in mutant embryos suggests that functional *DE*-, *B4U*- and *ENE*-RAREs are important in coordinating and maintaining the proper levels of nascent transcripts.

We examined whether changes in variance were correlated between different *Hoxb* genes and plotted ratios of average nascent transcript levels of one coding gene over a second coding gene (Fig. 5B) and found that the relative levels of coding genes were very close to those of wild-type in most RARE mutants. However, in the *DE*-RARE single mutant biphasic patterns of variation were observed for ratios of *Hoxb1*-*Hoxb9* and *Hoxb4*-*Hoxb9*, as indicated by the presence of two populations of abundance ratios for the coding genes in these mutants (Fig. 5B). Similarly, in the *DE-B4U-ENE* triple mutant, ratios of *Hoxb1*-*Hoxb4* and *Hoxb1*-*Hoxb9* displayed two distinct patterns. In *DE* mutants, these two populations can be attributed to the increased variance in *Hoxb1* nascent transcription (Fig. 4C), whereas in the triple mutant variations in both *Hoxb1* and *Hoxb9* contribute to the range of variability. These changes highlight that mutations in the RAREs can result in variable penetrance of their regulatory phenotypes, which suggests they contribute to regulation of both the levels and robustness in patterns of nascent transcription.

The level of regulatory variability in the RARE mutants may be greater than we have detected because large changes in coordinated *Hoxb* expression may affect development and viability of embryos. Mutations in the RAREs result in compensatory mechanisms altering levels of *Hoxb* transcripts that are often greater than wild-type (Figs 4 and 5). A limited range of expression thresholds may be compatible with viability and, through variable penetrance changes outside of this range, could result in lost embryos. In crosses of RAREs mutants we obtained reduced litter sizes for most homozygous RARE mutants compared with their heterozygous littermates, and all mutants have much lower numbers of viable animals born compared with the F1 wild-type animals (Fig. 5C, dashed red line). Homozygous animals often fail to breed and have to be backcrossed to wild-type animals to maintain viability of the lines. Therefore, there may be some selective pressure, whereby the most severely affected mutant embryos are eliminated or resorbed. This indicates that, although these RAREs are crucial for proper coordinated expression of *Hoxb* genes, other regulatory elements must have inputs that contribute to levels of *Hoxb* transcription that allow viable development.

### Mutations in RAREs change the spatial relationships between promoters in the *Hoxb* cluster

**Fig. 6.**
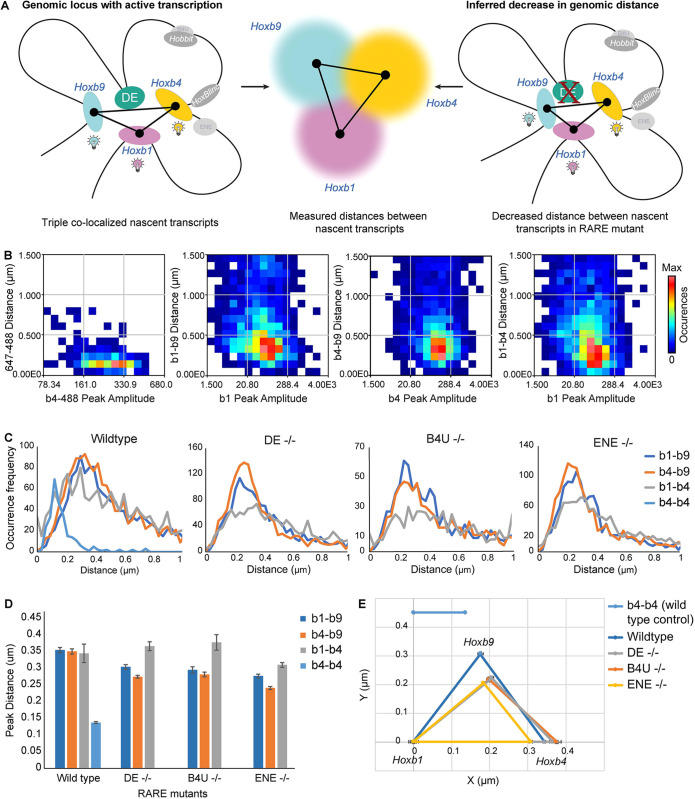
**Distances between triple co-localized nascent transcripts of coding genes is decreased in RARE mutants.** (A) Schematic to depict how distances are measured between triple co-localized nascent transcripts, where all three coding genes are being actively transcribed, and how the distance between transcripts is subsequently visualized in the triangle plot in E. (B) Distances between nascent spots (*y*-axis) compared with a peak reference (*x*-axis). First panel on the left represents distances from data using control *Hoxb4* intron and *Hoxb4* intron plus exon probes; each subsequent adjacent panel represents distances between *Hoxb1-Hoxb9*, *Hoxb4-Hoxb9* and *Hoxb1-Hoxb4*, respectively. The colors in the heat map indicate the frequency of occurrences of the nascent spots for corresponding distances. (C) Occurrence of nascent spots plotted against a gated distance of 0-1 μm. (D) Histogram for the peak values of the distance distributions between nascent spots. Colors of bars indicate which two gene spot distances are plotted, as indicated by key on the right. Data are mean±s.d. (E) Triangle plot for the peak distances observed between nascent spots of coding *Hoxb* genes in C. *Hoxb1* is anchored at 0, and relative distance changes between genes are plotted with respect to *Hoxb1*.

Recent experiments using smFISH and chromatin tracing in human cells have uncovered correlations between spatial coupling and co-bursting activities for genes in close proximity (Bohrer and Larson, 2022 preprint). They found that physical distance, not genomic distance, is a key factor driving transcriptional co-bursting between closely linked genes. To explore spatial relationships between promoters of active genes in our experiments, we examined the distances between co-localized spots of nascent transcription in data points where the *Hoxb1*, *Hoxb4* and *Hoxb9* coding genes were co-transcriptionally active in wild-type and the single RARE mutant embryos (Fig. 6A). We first corrected a spatial shift between the channels using a bead fiducial marker and applied this correction to all datasets. Then we identified the center of each nascent transcript spot and measured distances between the other co-localized spots (Fig. 6B). We then compared the distances between co-localized spots in RARE mutants versus wild-type to look for potential changes in the spatial relationships (Fig. 6A,C). We observed a range of distances between the co-localized spots and plotted the peak distance between spots (Fig. 6D). To aid in visualizing the changes in the spot distances we also generated a triangle plot (Fig. 6E).

To measure the threshold of resolution in our ability to distinguish distances between any two spots in wild-type cells, we used the corrected distance between the co-localized spots detected by the *Hoxb4* intron and exon probes (Fig. 2) as a control for each pairwise distance comparison. We found an average distance between the spots of 136 nm, with a simulated standard error of 1.5 nm. In all three pairwise combinations (*Hoxb1*-*Hoxb9*, *Hoxb1*-*Hoxb4* and *Hoxb4*-*Hoxb9*) despite differences in their genomic spacing, the spots of nascent transcripts are all separated by a mean distance of 350 nm (Fig. 6D,E). This distance is similar to that previously observed for co-transcriptionally regulated genes at comparable genomic distances in other systems (Chen et al., 2018; Benabdallah et al., 2019; Levo et al., 2022). It also is well within the 1100 nm physical space found to influence pairwise interactions between promoters of genes in close proximity when bursting (Bohrer and Larson, 2022 preprint).

The analysis in the single RARE mutants revealed that alterations in the RAREs changed the spatial relationships between promoters. The distances between nascent spots for *Hoxb1*-*Hoxb9* and *Hoxb4*-*Hoxb9* decreased, whereas there was no significant difference in the *Hoxb1*-*Hoxb4* distance (Fig. 6B-E). Thus, *Hoxb9* nascent spots are positioned closer to *Hoxb1* and *Hoxb4* in each of the single RARE mutants. It is interesting that this increased physical proximity correlates with increased ratios of *Hoxb1-Hoxb9* and *Hoxb4-Hoxb9* expression we observed in single RARE mutants, while the ratio for *Hoxb1-Hoxb4* expression does not change (Figs 4C,D and 5B). This is consistent with the idea that a mutation in the RAREs (enhancers) may alter the dynamics of regulatory interactions and physical distances between promoters during co-transcriptional activation of *Hoxb* genes.

## DISCUSSION

**Fig. 7.**
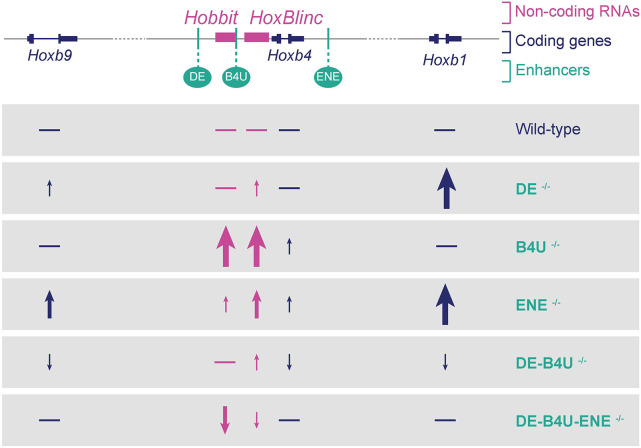
**Summary of effects of single and compound RARE mutants on patterns of nascent transcription.** Differences observed for coding and non-coding *Hoxb* genes in RARE mutants compared with wild-type are depicted. Arrows indicate direction of change, increase (up) or decrease (down), relative to wild-type expression. The thickness and height of each arrow indicates the relative levels of change, with the thickest and tallest arrows indicating the most significant changes. Horizontal lines indicate levels similar to wild-type.

In this study, we developed smFISH approaches with probes spanning introns to investigate how RA-dependent enhancers embedded in the *Hoxb* cluster regulate patterns of nascent transcription *in vivo* at the level of single cells within the developing mouse neural tube. We predominately detected nascent transcription of only a single *Hoxb* gene in each cell, with a much lower frequency of simultaneous transcription of one or two other transcriptional units. We found no evidence for simultaneous co-transcriptional coupling of all or specific subsets of genes in the cluster in individual cells. Analyses in embryos with single and/or compound mutations in RAREs of these enhancers revealed that each enhancer differentially impacts patterns of transcription within the cluster. The *DE* and *ENE* enhancers have a more global role in coordinating the levels and patterns of nascent transcription of genes in the cluster, whereas the *B4U* enhancer plays more of a local role impacting nascent transcription of near adjacent genes. Changes observed in compound mutants (summarized in Fig. 7) suggest that selectivity and competitive interactions between these enhancers plays an important role in coordinating and robustly maintaining the proper levels of nascent transcription of *Hoxb* genes. Together, these data suggest that rapid and dynamic enhancer-promoter interactions potentiate transcription of individual genes through combined inputs from these enhancers in coordinating the response to RA. This study provides new insight into the *in vivo* transcriptional dynamics of the *Hoxb* complex and raises interesting questions about the mechanisms and the roles of shared regulatory elements in coordinating expression.

Transcription in eukaryotes is thought to occur in bursts that are spaced out by refractory periods (Bartman et al., 2016; Fukaya et al., 2016). We are unable to infer transcriptional bursting rates because single transcripts are hard to separate from autofluorescence, but our data suggest that, at a given time, only one allele is usually transcriptionally active. We did not find evidence for the equivalent of a ‘topological operon’ (Levo et al., 2022) that co-regulates all *Hoxb* target genes through shared enhancers. We cannot exclude the possibility that there are sub-sets of genes in the cluster that are co-transcriptionally regulated, as we were unable to develop probe sets for all of the transcription units.

A surprising general finding in analysis of single RARE mutants is that we observed increased levels of nascent transcription of selected subsets of genes (Figs 4C,D and 5B), suggesting a change in the normal balance of regulatory interactions in the cluster. This implies that altering the activity of each of these enhancers impacts processes that regulate individual transcriptional bursting frequency or duration. Furthermore, in the *DE-B4U* double mutant, elevated levels of nascent transcripts per cell seen in the *DE* and *B4U* single mutants unexpectedly returned to near wild-type levels (Figs 4C and 5A). This suggests the presence of cross-talk (synergy, antagonism or competition) between the *DE* and *B4U* enhancers, which is altered in each single mutant but eliminated in the double mutant. This could arise through changes in enhancer-enhancer or enhancer-promoter interactions, or alterations in their individual or combined target preferences. The enhancers may also have optimal spatial proximities between each other and their promoters to potentiate activity. There is evidence that transcription can place constraints on chromatin around a gene (Bohrer and Larson, 2022 preprint), hence mutating an enhancer could alter transcription in a way that results in altered properties of chromatin and changes in gene expression.

Relevant to the increased levels of nascent transcription observed in single RARE mutants, we also observed changes in the spatial relationships between promoters in the cluster. *Hoxb9* nascent spots appear to be closer to *Hoxb1* and *Hoxb4* (Fig. 6) and this increased physical proximity correlates with increased patterns of expression. Finding that mutations in the RAREs of shared enhancers may alter the dynamics of regulatory interactions and physical distances between promoters raises the possibility that the *Hoxb* genomic locus may have a 3D architecture which can serve as a transcriptional hub during co-transcriptional activation of multiple *Hoxb* genes. Current Hi-C data do not provide sufficient resolution to robustly identify sub-TADs or regulatory hubs within the *Hoxb* TAD in the developing mouse neural tube. However, the pairwise distances between nascent spots for co-transcriptionally active *Hoxb1*, *Hoxb4* and *Hoxb9* was 350 nm, which is within the physical space (<1100 nm) that can influence interactions between promoters of genes in close proximity when bursting (Bohrer and Larson, 2022 preprint) and is consistent with models for transcriptional hubs or shared megadalton transcription factories (Muto et al., 2014; Cho et al., 2018; Furlong and Levine, 2018; Afzal and Krumlauf, 2022). In a hub the three RARE enhancers could be organized to dynamically compete or synergize with each other in interacting with and activating their target *Hoxb* promoters. This could potentiate the rapid turning on or off of nascent transcription of individual, and sometimes multiple genes, in a controlled manner.

In wild-type embryos, levels of nascent transcripts for the *Hoxb* genes were very reproducible and showed little variation within a narrow range, even between embryos (Figs 1F and 5A,B). However, in the single and compound RARE mutants there is a wide range of variation in levels of nascent transcripts for some, but not all, *Hoxb* genes. For example, in single *DE* and *B4U* mutants the range of expression of *Hoxb1* and *HoxBlinc* are much greater than wild-type, whereas in triple mutants there is a biphasic pattern in which levels can be well below or much greater than wild-type (Figs 4C,D and 5A,B). The significant changes in variance we observed in RARE mutant embryos suggests that functional *DE*-, *B4U*- and *ENE*-RAREs are important for controlling the degree of variability and coordinating proper levels of *Hoxb* nascent transcripts.

This study highlights the importance of these RAREs and shared enhancers in coordinating global levels of *Hoxb* expression in integrating the response to RA. Although essential for proper expression of *Hoxb* genes, other regulatory elements and/or mechanisms contribute to regulating patterns of transcription of these genes. For example, in the triple RARE mutant embryos we found a wide range in levels of nascent transcripts, from below to well above those of wild-type. Mutations of the RARE may enable new or altered inputs from other regulatory regions of the cluster that compensate for the loss in activity of these shared enhancers. In conclusion, our findings provide new insight into the roles of shared regulatory elements in coordinating regulation of the *in vivo* transcriptional dynamics of the *Hoxb* complex in mouse embryos.

## MATERIALS AND METHODS

### Mouse lines

Wild-type animals were generated from crosses of the Stowers F1 strain, which are a cross between CBA/CaJ×C57Bl/10J. The RARE enhancer mutants used for the project were generated by C.N. and Youngwook Ahn in the Krumlauf lab using a CRISPR approach. The *DE* mutant was generated as previously described (Ahn et al., 2014). For generation of the *B4U* and *ENE* single mutants, *DE-B4U* and *DE-B4U-ENE* compound mutants, gRNA sequences (Table S4) were ordered as oligonucleotides (Integrated DNA Technologies) with adjacent cloning sites added and cloned into *pX330* plasmids following directions provided by the Zhang Laboratory (Cong et al., 2013). The replacement oligonucleotide (at 75 ng/µl) and the Cas9 targeting plasmid (at 3 ng/µl) were combined and microinjected into the pronuclei of one-cell embryos. Two-cell embryos were transferred the following day into Stowers F1 recipient mice. All mutations introduced into the endogenous *Hoxb* locus were validated by sequencing to ensure that the specific base pair mutations were properly generated. All animals were maintained by Laboratory Animal Services (LAS) at Stowers Institute for Medical Research (SIMR) and all experiments involving mice were approved by the Institutional Animal Care and Use Committee (IACUC) of the Stowers Institute for Medical Research under a protocol (Protocol 2021-134) issued to R.K. All RARE mutant lines generated and used in this study are preserved through cryopreservation of sperm and freely available upon request to R.K.

### Genotyping of animals

To genotype the animals, tail samples were collected after weaning the animals. The tails were either processed internally and genotyped or directly sent for processing to Transnetyx. For each mutant animal, validated Transnetyx assays were set up to detect the presence of wild-type or mutant DNA sequences.

### Staging of embryos

For each embryo collected, the number of somites were counted to ensure the same developmental age was used for all experiments. Embryos were considered to be 9.5 dpc if they had anywhere from 21 to 29 somite pairs, but for this paper, wild-type and mutant RARE embryos containing 24 somite pairs were used for all experiments.

### Collection and fixing of embryo samples

For collecting wild-type embryos, Stowers F1 animals were paired and pregnant females (upon plug checking) were marked for embryo collection at 9.5 dpc. For enhancer mutants, mouse lines were bred to homozygosity. The homozygous animals were then crossed, and pregnant females (upon plug checking) were marked for embryo collection at 9.5 dpc. The pregnant females were euthanized according to IACUC protocol number 2021-134. The uterine tissue containing embryo sacs was extracted from the abdominal cavity and kept in ice-cold PBS solution. After collecting all 9.5 dpc embryos in ice-cold PBS, the embryos were transferred to 4% paraformaldehyde solution and kept overnight at 4°C. The embryos were then dehydrated using methanol gradients of 25%, 50%, 75% and 100%. The embryos were stored at −20°C for at least 1 day before histological processing.

### Histological preparation of embryos

For smFISH, RNAase free conditions were used. The embryos were kept in methanol and rehydrated slowly with PBS. The embryos were cut into three sections to aid with correct orientations; head (cut made after brachial arches), mid (cut made after hindlimb bud) and tail sections. After this, the samples were dehydrated using a 15% solution of sucrose made in DEPC-treated PBS. Once the samples were saturated, they were embedded into OCT compound (VWR, 25608-930) using the Histochill unit (SP Scientific Products, HC80). Using a Thermo Fisher Scientific Microm Cryostar NX70 cryostat, transverse sections at 10 μm were placed on Sure Bond charged slides (Avantik, SL6332-1); alternate sections were placed onto a single slide. The slides were stored at −80°C if not going to be used within the same week, or kept at −20°C if going to be used within the same week.

### Probe design

For probe design, genomic sequences were obtained from Genome Browser (https://genome.ucsc.edu/). The multiple 20-22 bp intronic and exonic probes (for *Hoxb4*, *Hobbit* and *HoxBlinc*) and the multiple 29 bp intronic probe (for *Hoxb9*) were designed using the Stellaris Probe generator from LGC Biosearch Technologies (https://www.biosearchtech.com/support/tools/design-software/stellaris-probe-designer). The *Hoxb1* and *Gapdh* mouse probes were brought pre-labeled from the Stellaris website.

### Probe synthesis

For smFISH, the probe-set sequences were either bought from the Design Ready version offered by Stellaris or were designed using the Stellaris probe designer tool. For probe labeling, unlabeled probe sets carrying a C-term TEG-Amino tag, were purchased from Biosearch Technologies and labeled as previously described (De Kumar et al., 2017). Probes (5 nmol) were fluorescently labeled overnight in 0.1 M sodium tetraborate (pH 9) at 4°C. Labeling occurred with AlexaFluor-488, AlexaFluor-568 or AlexaFluor-647. Two units of amine-reactive succinimidyl ester Decapacks (Thermo Fisher Scientific) were used for each reaction and, following quenching, labeled probes were purified using reverse-phase HPLC. Probes were separated using an Ettan LC (GE Healthcare) using a 4.6×250-mm, 5-μm, C18-EMS end-capped Kinetex column (Phenomenex). Mobile phase A was 0.1 M ammonium acetate (EMD Millipore) (pH 7) and mobile phase B was acetonitrile (Millipore). A linear gradient of 5% B to 100% (vol/vol) B was run over the course of 20 min at 1 ml/min. Peaks were monitored at 280 nm for probe and at 488, 568 or 647 nm, depending on the dye. Dual positive peaks were collected by hand and concentrated by spin vac. Samples were then resuspended in DEPC water to a final volume of 100 µl. See Table S1 for details of all probes and their fluorophores.

### smFISH optimized protocol

The smFISH existing protocol (Raj and Tyagi, 2010) was optimized and adopted for use with mouse embryo sections. Tissue slides were thawed at room temperature for ∼10 min. The samples were permeabilized with 2 ng/ml concentration of proteinase K in PBS for 10 min. The sample was kept in wash buffer A (Stellaris, SMF-WA1-60) until the hybridization mixture was prepared from 100 μl of deionized Formamide (VWR, CAS-75-12-7) and 900 μl of hybridization buffer (Stellaris, SMF-HB1-10). For every 100 μl of hybridization mixture, 1 μl of probe was added (∼5 nmol stock probe dissolved in 100 μl of nuclease free water), and up to three probes were added to each mixture and put on tissue slides. The slides were covered with a coverslip, heated at 65°C for 10 min and kept in a wet chamber overnight at 30°C (∼16 h). Tissue slides were then washed with wash buffer A for 30 min at 37°C. DAPI (1:5000) was added to wash buffer A, and slides were kept in this mixture at room temperature for 10 min followed by 2-4 h at 4°C. Slides were washed with wash buffer B (Stellaris, SMF-WB1-20) for 15 min at room temperature. Slides were mounted with ProLong Gold Antifade Mountant (Thermo Fisher Scientific), covered with a 0.13-0.16 mm long coverslip and dried at room temperature before imaging.

### Imaging with Nikon spinning disk

Tissue sections on the slides were imaged using the Nikon Ti-E microscope coupled to a Yokogawa CSU-W1 spinning disk using the Hamamatsu Flash 4 sCMOS camera. The images were captured using 60× Plan Apochromat (NA 1.4) oil objective. The sections to image were manually identified using a 10× objective (NA 1.45). The images were obtained at 100% laser power for far red 633 nm, red 561 nm, green 488 nm and DAPI 405 nm lasers. For each channel the following filters were used: DAPI, ET455/50m; green, ET525/36m; red, ET605/70m; far red, ET700/75m. A Nikon Elements Job ‘Tiler’ was used to capture the images if tiles were taken, and the image was stitched later in Fiji (https://imagej.net/software/fiji) using the grid/collection stitching plugin. The order of experiments was Lambda (*z*-series), so each color was imaged in *z* before moving to the next color. For obtaining a *z*-stack through the tissue section, each slice imaged was 1 µm apart and a range of 20 steps was taken. Images were obtained in the order of 633 (for the 647 probe), 561 (for the 555 probe), 488 (for the atto 488 probe) and 405 (for DAPI). On a slide, the whole tissue section was imaged, and for each slide a single row was imaged all the way across for each genotype of embryo. All images were stitched using Fiji before further processing.

### Deep learning processing on images

RNAFish spots were segmented using DeepFiji (Nuckolls et al., 2020). In brief, a small subset of spots from different probes was manually annotated in Fiji and used to train a 2D Unet (Ronneberger et al., 2015) model. This model was used to infer spot probabilities from full image sets. After obtaining the results from DeepFiji, 3D image sets were thresholded and 3D segmented in order to apply a size filter, and then projected to 2D. Combinations of these projections were examined to find cases where spots had overlaps in multiple channels. Original spots and overlapped spots were then reduced to a single pixel and blurred to provide heat maps. Spot locations were also saved for future analysis. The relevant code for preparing image files for DeepFiji, and the post-processing afterwards, can be found at https://github.com/jouyun/smc-macros.

### Spot fitting

To be able to see nascent spots detected over the whole tissue section without having to zoom into the image, the nascent spots localization file was used to make a spot file. To generate a spot file for this visual representation, a python program was used (https://github.com/cwood1967/afzal_z_dev2023). The spot file could be edited in Fiji, such that the sizes of the individual spots could be increased. Spot size for each channel was increased consistently across all samples, and this file was overlaid onto DAPI.

### Distance measurement methods

Distances were measured between smFISH spots via Gaussian fitting with and without color correction, using custom plugins written for Fiji (available at https://research.stowers.org/imagejplugins). Although color alignment errors are negligible for measurement of co-localization, they become significant when considering nanoscale genomic locus arrangements. For such measurements, color correction parameters were determined by fitting tetraspeck beads (Thermo Fisher Scientific) to Gaussian functions and finding the translation, rotation and scaling parameters that minimize the distances between peaks in different channels. Those same transformations were applied to smFISH images collected with the same parameters. After color correction, images were pre-processed via Gaussian blurring with a standard deviation of 1 pixel and rolling ball background subtraction with a ball radius of 15 pixels. Peaks were then found in the far-red channel (due to its low background) by a ‘max not mask’ (spot finding Fiji plugin) approach. Briefly, the maximum voxel in the image is repeatedly found and then a spheroid around that voxel is masked with zeros. For this analysis, the spheroid diameter was 20 pixels in *xy* and six slices in *z*. This procedure is repeated until no other voxels are found with an intensity above 5% of the image maximum.

Once positions were found, they were refined by non-linear least squares 3D Gaussian fitting of a 20×20 pixel stack of six slices surrounding the potential position in all three channels. Best fit peak positions were constrained to be within two pixels distance in *xy* and two slices in *z* of the maximum position found above. For each pair of probes, 2D histograms of spot amplitude versus spot distance were created (see Fig. 6) and the area surrounding the main peak of this histogram extending from distances of 0-1 μm was gated and plotted in Fig. 6E. Finally, that histogram was fit to a one-dimensional Gaussian, again by non-linear least squares, to obtain the peak position of the distance distribution.

### Measurements and analysis post-DL on images

Images were processed using Fiji. Using max projection from the DAPI channel, the regions of interest (ROIs) were marked in each tissue section. ROIs for the neural tube and adjacent somites were marked for each tail section by hand in the DAPI channel. Measurements were made for the total intensity of nascent transcripts for each ROI, and from each ROI the background intensity was subtracted. For each ROI, a custom in-house written plugin ‘filter table spatial ROI jru v1’ was used to also extract the exact count of nascent spots and their co-localizations from the DL processing excel file output for every nascent spot over the whole tissue. To make bar plots of exact number of nascent transcripts for each condition – wild-type or RARE mutant – an average of 6-8 tail sections was plotted.

### Cell counts for each ROI

In the neural tube, the cells are densely packed together, which makes it very hard to automate their counting. Conventional and Deep Learned algorithms have a hard time separating out cells and, even manually, it is difficult to go through each *z*-slice and count. To counter this problem, a sum projection file was generated using the DAPI channel for each tissue section. A small region in which there was a lower density of cells was outlined, cells in that region were counted, and the integrated DAPI intensity per nuclei for this region was made. A ratio of integrated DAPI intensity/cell was used to roughly estimate the number of cells in the ROIs marked in each section (integrated DAPI intensity of each ROI divided by the ratio of integrated DAPI intensity/cell, to equal the number of cells present within the ROI). This was carried out for all tail sections in all wild-type and RARE mutants. For each tissue section, the exact number of nascent transcripts was divided by the corresponding cell counts for each ROI, and these values were used to plot the boxplots for the exact number of nascent transcripts per cell count.

### Statistical analysis of data

The nascent transcripts datasets were assessed for normality, and it was observed that the samples were bell-shaped. Therefore, the non-parametric test Mann–Whitney (also called Wilcoxon rank-sum) test was performed, and all RARE mutant samples were compared against wild-type for each probe set. Asterisks denote significance. To calculate how the range in nascent transcript expression was changing for each gene, variance was calculated using a parametric F-test. Any significant change in variance (*P*-value<0.05) compared against wild-type samples was denoted by a star.

## Supplementary Material

10.1242/develop.201259_sup1 Supplementary information

## People behind the papers

This article has an associated People behind the papers interview with some of the authors.

## References

[DEV201259C1] Afzal, Z. and Krumlauf, R. (2022). Transcriptional regulation and implications for controlling Hox gene expression. *J. Dev. Biol.* 10, 4. 10.3390/jdb10010004 35076545 PMC8788451

[DEV201259C2] Ahn, Y., Mullan, H. E. and Krumlauf, R. (2014). Long-range regulation by shared retinoic acid response elements modulates dynamic expression of posterior Hoxb genes in CNS development. *Dev. Biol.* 388, 134-144. 10.1016/j.ydbio.2014.01.027 24525295

[DEV201259C3] Andrey, G., Montavon, T., Mascrez, B., Gonzalez, F., Noordermeer, D., Leleu, M., Trono, D., Spitz, F. and Duboule, D. (2013). A switch between topological domains underlies HoxD genes collinearity in mouse limbs. *Science* 340, 1234167. 10.1126/science.1234167 23744951

[DEV201259C4] Arendt, D. (2018). Hox genes and body segmentation. *Science* 361, 1310-1311. 10.1126/science.aav0692 30262483

[DEV201259C5] Balkaschina, E. I. (1929). Ein Fall der Erbhomeosis (die Genovarition“aristopedia”) bei Drosophila melanogaster. *Wilhelm Roux Arch. Entwickl. Mech. Org.* 115, 448-463. 10.1007/BF02079002 28353870

[DEV201259C6] Bartman, C. R., Hsu, S. C., Hsiung, C. C.-S., Raj, A. and Blobel, G. A. (2016). Enhancer regulation of transcriptional bursting parameters revealed by forced chromatin looping. *Mol. Cell* 62, 237-247. 10.1016/j.molcel.2016.03.007 27067601 PMC4842148

[DEV201259C7] Batut, P. J., Bing, X. Y., Sisco, Z., Raimundo, J., Levo, M. and Levine, M. S. (2022). Genome organization controls transcriptional dynamics during development. *Science* 375, 566-570. 10.1126/science.abi7178 35113722 PMC10368186

[DEV201259C8] Bel-Vialar, S., Itasaki, N. and Krumlauf, R. (2002). Initiating Hox gene expression: in the early chick neural tube differential sensitivity to FGF and RA signaling subdivides the HoxB genes in two distinct groups. *Development* 129, 5103-5115. 10.1242/dev.129.22.5103 12399303

[DEV201259C9] Benabdallah, N. S., Williamson, I., Illingworth, R. S., Kane, L., Boyle, S., Sengupta, D., Grimes, G. R., Therizols, P. and Bickmore, W. A. (2019). Decreased enhancer-promoter proximity accompanying enhancer activation. *Mol. Cell* 76, 473-484.e7. 10.1016/j.molcel.2019.07.038 31494034 PMC6838673

[DEV201259C10] Berlivet, S., Paquette, D., Dumouchel, A., Langlais, D., Dostie, J. and Kmita, M. (2013). Clustering of tissue-specific sub-TADs accompanies the regulation of HoxA genes in developing limbs. *PLoS Genet.* 9, e1004018. 10.1371/journal.pgen.1004018 24385922 PMC3873244

[DEV201259C11] Berrocal, A., Lammers, N. C., Garcia, H. G. and Eisen, M. B. (2020). Kinetic sculpting of the seven stripes of the Drosophila even-skipped gene. *eLife* 9, e61635. 10.7554/eLife.61635 33300492 PMC7864633

[DEV201259C12] Bertrand, N., Roux, M., Ryckebüsch, L., Niederreither, K., Dollé, P., Moon, A., Capecchi, M. and Zaffran, S. (2011). Hox genes define distinct progenitor sub-domains within the second heart field. *Dev. Biol.* 353, 266-274. 10.1016/j.ydbio.2011.02.029 21385575 PMC3115524

[DEV201259C13] Bohrer, C. H. and Larson, D. R. (2023). Synthetic analysis of chromatin tracing and live-cell imaging indicates pervasive spatial coupling between genes. *eLife* 12, e81861. 10.7554/eLife.81861 36790144 PMC9984193

[DEV201259C14] Bothma, J. P., Garcia, H. G., Esposito, E., Schlissel, G., Gregor, T. and Levine, M. (2014). Dynamic regulation of eve stripe 2 expression reveals transcriptional bursts in living Drosophila embryos. *Proc. Natl. Acad. Sci. USA* 111, 10598-10603. 10.1073/pnas.1410022111 24994903 PMC4115566

[DEV201259C15] Brend, T., Gilthorpe, J., Summerbell, D. and Rigby, P. W. J. (2003). Multiple levels of transcriptional and post-transcriptional regulation are required to define the domain of Hoxb4 expression. *Development* 130, 2717-2728. 10.1242/dev.00471 12736215

[DEV201259C16] Bridges, C. B. and Dobzhan, T. (1933). The mutant “proboscipedia” in Drosophila melanogaster - a case of hereditary homeosis. *Wilhelm. Roux’ Arch. Entwicklungsmech. Org.* 127, 575-590. 10.1007/BF01380474 28354344

[DEV201259C17] Carroll, S. B. (1995). Homeotic genes and the evolution of arthropods and chordates. *Nature* 376, 479-485. 10.1038/376479a0 7637779

[DEV201259C18] Chambeyron, S. and Bickmore, W. A. (2004). Chromatin decondensation and nuclear reorganization of the HoxB locus upon induction of transcription. *Genes Dev.* 18, 1119-1130. 10.1101/gad.292104 15155579 PMC415637

[DEV201259C19] Chen, H., Levo, M., Barinov, L., Fujioka, M., Jaynes, J. B. and Gregor, T. (2018). Dynamic interplay between enhancer-promoter topology and gene activity. *Nat. Genet.* 50, 1296-1303. 10.1038/s41588-018-0175-z 30038397 PMC6119122

[DEV201259C20] Cho, W.-K., Spille, J.-H., Hecht, M., Lee, C., Li, C., Grube, V. and Cisse, I. I. (2018). Mediator and RNA polymerase II clusters associate in transcription-dependent condensates. *Science* 361, 412-415. 10.1126/science.aar4199 29930094 PMC6543815

[DEV201259C21] Choi, J., Lysakovskaia, K., Stik, G., Demel, C., Söding, J., Tian, T. V., Graf, T. and Cramer, P. (2021). Evidence for additive and synergistic action of mammalian enhancers during cell fate determination. *eLife* 10, e65381. 10.7554/eLife.65381 33770473 PMC8004103

[DEV201259C22] Cong, L., Ran, F. A., Cox, D., Lin, S., Barretto, R., Habib, N., Hsu, P. D., Wu, X., Jiang, W., Marraffini, L. A. et al. (2013). Multiplex genome engineering using CRISPR/Cas systems. *Science* 339, 819-823. 10.1126/science.1231143 23287718 PMC3795411

[DEV201259C23] Crocker, J., Abe, N., Rinaldi, L., McGregor, A. P., Frankel, N., Wang, S., Alsawadi, A., Valenti, P., Plaza, S., Payre, F. et al. (2015). Low affinity binding site clusters confer hox specificity and regulatory robustness. *Cell* 160, 191-203. 10.1016/j.cell.2014.11.041 25557079 PMC4449256

[DEV201259C24] Darras, S., Fritzenwanker, J. H., Uhlinger, K. R., Farrelly, E., Pani, A. M., Hurley, I. A., Norris, R. P., Osovitz, M., Terasaki, M., Wu, M. et al. (2018). Anteroposterior axis patterning by early canonical Wnt signaling during hemichordate development. *PLoS Biol.* 16, e2003698. 10.1371/journal.pbio.2003698 29337984 PMC5786327

[DEV201259C25] De Kumar, B. and Krumlauf, R. (2016). HOXs and lincRNAs: two sides of the same coin. *Sci. Adv.* 2, e1501402. 10.1126/sciadv.1501402 27034976 PMC4805430

[DEV201259C26] De Kumar, B., Parrish, M. E., Slaughter, B. D., Unruh, J. R., Gogol, M., Seidel, C., Paulson, A., Li, H., Gaudenz, K., Peak, A. et al. (2015). Analysis of dynamic changes in retinoid-induced transcription and epigenetic profiles of murine Hox clusters in ES cells. *Genome Res.* 25, 1229-1243. 10.1101/gr.184978.114 26025802 PMC4510006

[DEV201259C27] De Kumar, B., Parker, H. J., Parrish, M. E., Lange, J. J., Slaughter, B. D., Unruh, J. R., Paulson, A. and Krumlauf, R. (2017). Dynamic regulation of Nanog and stem cell-signaling pathways by Hoxa1 during early neuro-ectodermal differentiation of ES cells. *Proc. Natl. Acad. Sci. U.S.A.* 114, 5838-5845. 10.1073/pnas.1610612114 28584089 PMC5468655

[DEV201259C28] de Laat, W. and Duboule, D. (2013). Topology of mammalian developmental enhancers and their regulatory landscapes. *Nature* 502, 499-506. 10.1038/nature12753 24153303

[DEV201259C29] Degani, N., Lubelsky, Y., Perry, R. B.-T., Ainbinder, E. and Ulitsky, I. (2021). Highly conserved and cis-acting lncRNAs produced from paralogous regions in the center of HOXA and HOXB clusters in the endoderm lineage. *PLoS Genet.* 17, e1009681. 10.1371/journal.pgen.1009681 34280202 PMC8330917

[DEV201259C30] Delpretti, S., Montavon, T., Leleu, M., Joye, E., Tzika, A., Milinkovitch, M. and Duboule, D. (2013). Multiple enhancers regulate Hoxd genes and the Hotdog LncRNA during cecum budding. *Cell Rep.* 5, 137-150. 10.1016/j.celrep.2013.09.002 24075990

[DEV201259C31] Deng, W., Lee, J., Wang, H., Miller, J., Reik, A., Gregory, P. D., Dean, A. and Blobel, G. A. (2012). Controlling long-range genomic interactions at a native locus by targeted tethering of a looping factor. *Cell* 149, 1233-1244. 10.1016/j.cell.2012.03.051 22682246 PMC3372860

[DEV201259C32] Deng, C., Li, Y., Zhou, L., Cho, J., Patel, B., Terada, N., Li, Y., Bungert, J., Qiu, Y. and Huang, S. (2016). HoxBlinc RNA recruits Set1/MLL complexes to activate Hox gene expression patterns and mesoderm lineage development. *Cell Rep.* 14, 103-114. 10.1016/j.celrep.2015.12.007 26725110 PMC4706800

[DEV201259C33] Deschamps, J. and Duboule, D. (2017). Embryonic timing, axial stem cells, chromatin dynamics, and the Hox clock. *Genes Dev.* 31, 1406-1416. 10.1101/gad.303123.117 28860158 PMC5588924

[DEV201259C34] Deschamps, J. and van Nes, J. (2005). Developmental regulation of the Hox genes during axial morphogenesis in the mouse. *Development* 132, 2931-2942. 10.1242/dev.01897 15944185

[DEV201259C35] Diez del Corral, R. and Storey, K. G. (2004). Opposing FGF and retinoid pathways: a signalling switch that controls differentiation and patterning onset in the extending vertebrate body axis. *BioEssays* 26, 857-869. 10.1002/bies.20080 15273988

[DEV201259C36] Dixon, J. R., Selvaraj, S., Yue, F., Kim, A., Li, Y., Shen, Y., Hu, M., Liu, J. S. and Ren, B. (2012). Topological domains in mammalian genomes identified by analysis of chromatin interactions. *Nature* 485, 376-380. 10.1038/nature11082 22495300 PMC3356448

[DEV201259C37] Duboule, D. and Dollé, P. (1989). The structural and functional organization of the murine *HOX* gene family resembles that of *Drosophila* homeotic genes. *EMBO J.* 8, 1497-1505. 10.1002/j.1460-2075.1989.tb03534.x 2569969 PMC400980

[DEV201259C38] Dunn, T. M., Hahn, S., Ogden, S. and Schleif, R. F. (1984). An operator at −280 base pairs that is required for repression of araBAD operon promoter: addition of DNA helical turns between the operator and promoter cyclically hinders repression. *Proc. Natl. Acad. Sci. USA* 81, 5017-5020. 10.1073/pnas.81.16.5017 6089170 PMC391628

[DEV201259C39] Dupé, V., Davenne, M., Brocard, J., Dollé, P., Mark, M., Dierich, A., Chambon, P. and Rijli, F. M. (1997). In vivo functional analysis of the Hoxa-1 3′ retinoic acid response element (3′RARE). *Development* 124, 399-410. 10.1242/dev.124.2.399 9053316

[DEV201259C40] Eck, E., Liu, J., Kazemzadeh-Atoufi, M., Ghoreishi, S., Blythe, S. A. and Garcia, H. G. (2020). Quantitative dissection of transcription in development yields evidence for transcription-factor-driven chromatin accessibility. *eLife* 9, e56429. 10.7554/eLife.56429 33074101 PMC7738189

[DEV201259C41] Folberg, A., Nagy Kovacs, E., Luo, J., Giguere, V. and Featherstone, M. S. (1999). RARbeta mediates the response of Hoxd4 and Hoxb4 to exogenous retinoic acid. *Dev. Dyn.* 215, 96-107. 10.1002/(SICI)1097-0177(199906)215:2<96::AID-DVDY2>3.0.CO;2-T 10373014

[DEV201259C42] Frank, D. and Sela-Donenfeld, D. (2019). Hindbrain induction and patterning during early vertebrate development. *Cell. Mol. Life Sci.* 76, 941-960. 10.1007/s00018-018-2974-x 30519881 PMC11105337

[DEV201259C43] Fukaya, T., Lim, B. and Levine, M. (2016). Enhancer control of transcriptional bursting. *Cell* 166, 358-368. 10.1016/j.cell.2016.05.025 27293191 PMC4970759

[DEV201259C44] Furlong, E. E. M. and Levine, M. (2018). Developmental enhancers and chromosome topology. *Science* 361, 1341-1345. 10.1126/science.aau0320 30262496 PMC6986801

[DEV201259C45] Garber, R. L., Kuroiwa, A. and Gehring, W. J. (1983). Genomic and cDNA clones of the homeotic locus Antennapedia in Drosophila. *EMBO J.* 2, 2027-2036. 10.1002/j.1460-2075.1983.tb01696.x 6416827 PMC555405

[DEV201259C46] Gaskill, M. and Harrison, M. (2022). Tethering gene regulation to chromatin organization. *Science* 375, 491-492. 10.1126/science.abn6380 35113712

[DEV201259C47] Gentile, C. and Kmita, M. (2018). The remote transcriptional control of Hox genes. *Int. J. Dev. Biol.* 62, 685-692. 10.1387/ijdb.180198mk 30604838

[DEV201259C48] Goh, K. L., Yang, J. T. and Hynes, R. O. (1997). Mesodermal defects and cranial neural crest apoptosis in α5 integrin-null embryos. *Development* 124, 4309-4319. 10.1242/dev.124.21.4309 9334279

[DEV201259C49] Gonzalez, F., Duboule, D. and Spitz, F. (2007). Transgenic analysis of Hoxd gene regulation during digit development. *Dev. Biol.* 306, 847-859. 10.1016/j.ydbio.2007.03.020 17448461

[DEV201259C50] Gould, A., Morrison, A., Sproat, G., White, R. A. and Krumlauf, R. (1997). Positive cross-regulation and enhancer sharing: two mechanisms for specifying overlapping *Hox* expression patterns. *Genes Dev.* 11, 900-913. 10.1101/gad.11.7.900 9106661

[DEV201259C51] Gould, A., Itasaki, N. and Krumlauf, R. (1998). Initiation of rhombomeric *Hoxb4* expression requires induction by somites and a retinoid pathway. *Neuron* 21, 39-51. 10.1016/S0896-6273(00)80513-9 9697850

[DEV201259C52] Graham, A., Papalopulu, N. and Krumlauf, R. (1989). The murine and *Drosophila* homeobox gene complexes have common features of organization and expression. *Cell* 57, 367-378. 10.1016/0092-8674(89)90912-4 2566383

[DEV201259C53] Gregor, T., Garcia, H. G. and Little, S. C. (2014). The embryo as a laboratory: quantifying transcription in Drosophila. *Trends Genet.* 30, 364-375. 10.1016/j.tig.2014.06.002 25005921 PMC4129518

[DEV201259C54] Hafen, E., Levine, M. and Gehring, W. J. (1984). Regulation of Antennapedia transcript distribution by the bithorax complex in Drosophila. *Nature* 307, 287-289. 10.1038/307287a0 6420705

[DEV201259C55] He, S., Del Viso, F., Chen, C.-Y., Ikmi, A., Kroesen, A. E. and Gibson, M. C. (2018). An axial Hox code controls tissue segmentation and body patterning in Nematostella vectensis. *Science* 361, 1377-1380. 10.1126/science.aar8384 30262503

[DEV201259C56] Heinz, S., Romanoski, C. E., Benner, C. and Glass, C. K. (2015). The selection and function of cell type-specific enhancers. *Nat. Rev. Mol. Cell Biol.* 16, 144-154. 10.1038/nrm3949 25650801 PMC4517609

[DEV201259C57] Henriques, T., Scruggs, B. S., Inouye, M. O., Muse, G. W., Williams, L. H., Burkholder, A. B., Lavender, C. A., Fargo, D. C. and Adelman, K. (2018). Widespread transcriptional pausing and elongation control at enhancers. *Genes Dev.* 32, 26-41. 10.1101/gad.309351.117 29378787 PMC5828392

[DEV201259C58] Houle, M., Sylvestre, J.-R. and Lohnes, D. (2003). Retinoic acid regulates a subset of Cdx1 function in vivo. *Development* 130, 6555-6567. 10.1242/dev.00889 14660544

[DEV201259C59] Ke, Y., Xu, Y., Chen, X., Feng, S., Liu, Z., Sun, Y., Yao, X., Li, F., Zhu, W., Gao, L. et al. (2017). 3D chromatin structures of mature gametes and structural reprogramming during mammalian embryogenesis. *Cell* 170, 367-381.e20. 10.1016/j.cell.2017.06.029 28709003

[DEV201259C60] Kong, S., Bohl, D., Li, C. and Tuan, D. (1997). Transcription of the HS2 enhancer toward a cis-linked gene is independent of the orientation, position, and distance of the enhancer relative to the gene. *Mol. Cell. Biol.* 17, 3955-3965. 10.1128/MCB.17.7.3955 9199330 PMC232248

[DEV201259C61] Kreibich, E., Kleinendorst, R., Barzaghi, G., Kaspar, S. and Krebs, A. R. (2022). *Title*. Cold Spring Harbor Laboratory.

[DEV201259C62] Krumlauf, R. and Wilkinson, D. G. (2021). Segmentation and patterning of the vertebrate hindbrain. *Development* 148, dev186460. 10.1242/dev.186460 34323269 PMC7611710

[DEV201259C63] Levo, M., Raimundo, J., Bing, X. Y., Sisco, Z., Batut, P. J., Ryabichko, S., Gregor, T. and Levine, M. S. (2022). Transcriptional coupling of distant regulatory genes in living embryos. *Nature* 605, 754-760. 10.1038/s41586-022-04680-7 35508662 PMC9886134

[DEV201259C64] Lewis, E. B. (1978). A gene complex controlling segmentation in Drosophila. *Nature* 276, 565-570. 10.1038/276565a0 103000

[DEV201259C65] Liu, Z. and Tjian, R. (2018). Visualizing transcription factor dynamics in living cells. *J. Cell Biol.* 217, 1181-1191. 10.1083/jcb.201710038 29378780 PMC5881510

[DEV201259C66] Long, H. K., Prescott, S. L. and Wysocka, J. (2016). Ever-changing landscapes: transcriptional enhancers in development and evolution. *Cell* 167, 1170-1187. 10.1016/j.cell.2016.09.018 27863239 PMC5123704

[DEV201259C67] Maconochie, M., Nonchev, S., Morrison, A. and Krumlauf, R. (1996). Paralogous *Hox* genes: function and regulation. *Annu. Rev. Genet.* 30, 529-556. 10.1146/annurev.genet.30.1.529 8982464

[DEV201259C68] Maconochie, M. K., Nonchev, S., Studer, M., Chan, S. K., Pöpperl, H., Sham, M. H., Mann, R. S. and Krumlauf, R. (1997). Cross-regulation in the mouse *HoxB* complex: the expression of *Hoxb2* in rhombomere 4 is regulated by *Hoxb1*. *Genes Dev.* 11, 1885-1896. 10.1101/gad.11.14.1885 9242495

[DEV201259C69] Mallo, M., Wellik, D. M. and Deschamps, J. (2010). Hox genes and regional patterning of the vertebrate body plan. *Dev. Biol.* 344, 7-15. 10.1016/j.ydbio.2010.04.024 20435029 PMC2909379

[DEV201259C70] Marshall, H., Studer, M., Pöpperl, H., Aparicio, S., Kuroiwa, A., Brenner, S. and Krumlauf, R. (1994). A conserved retinoic acid response element required for early expression of the homeobox gene *Hoxb-*1. *Nature* 370, 567-571. 10.1038/370567a0 7914354

[DEV201259C71] Martin, A., Serano, J. M., Jarvis, E., Bruce, H. S., Wang, J., Ray, S., Barker, C. A., O'Connell, L. C. and Patel, N. H. (2016). CRISPR/Cas9 mutagenesis reveals versatile roles of Hox genes in crustacean limb specification and evolution. *Curr. Biol.* 26, 14-26. 10.1016/j.cub.2015.11.021 26687626

[DEV201259C72] Mazzoni, E. O., Mahony, S., Peljto, M., Patel, T., Thornton, S. R., McCuine, S., Reeder, C., Boyer, L. A., Young, R. A., Gifford, D. K. et al. (2013). Saltatory remodeling of Hox chromatin in response to rostrocaudal patterning signals. *Nat. Neurosci.* 16, 1191-1198. 10.1038/nn.3490 23955559 PMC3799941

[DEV201259C73] McGinnis, W. and Krumlauf, R. (1992). Homeobox genes and axial patterning. *Cell* 68, 283-302. 10.1016/0092-8674(92)90471-N 1346368

[DEV201259C74] Medina-Martínez, O. and Ramírez-Solis, R. (2003). In vivo mutagenesis of the Hoxb8 hexapeptide domain leads to dominant homeotic transformations that mimic the loss-of-function mutations in genes of the Hoxb cluster. *Dev. Biol.* 264, 77-90. 10.1016/j.ydbio.2003.07.020 14623233

[DEV201259C75] Merrill, V. K., Diederich, R. J., Turner, F. R. and Kaufman, T. C. (1989). A genetic and developmental analysis of mutations in labial, a gene necessary for proper head formation in Drosophila melanogaster. *Dev. Biol.* 135, 376-391. 10.1016/0012-1606(89)90187-5 2570723

[DEV201259C76] Mir, M., Stadler, M. R., Ortiz, S. A., Hannon, C. E., Harrison, M. M., Darzacq, X. and Eisen, M. B. (2018). Dynamic multifactor hubs interact transiently with sites of active transcription in Drosophila embryos. *eLife* 7, e40497. 10.7554/eLife.40497 30589412 PMC6307861

[DEV201259C77] Morcillo, P., Rosen, C., Baylies, M. K. and Dorsett, D. (1997). Chip, a widely expressed chromosomal protein required for segmentation and activity of a remote wing margin enhancer in Drosophila. *Genes Dev.* 11, 2729-2740. 10.1101/gad.11.20.2729 9334334 PMC316608

[DEV201259C78] Muto, A., Ikeda, S., Lopez-Burks, M. E., Kikuchi, Y., Calof, A. L., Lander, A. D. and Schilling, T. F. (2014). Nipbl and mediator cooperatively regulate gene expression to control limb development. *PLoS Genet.* 10, e1004671. 10.1371/journal.pgen.1004671 25255084 PMC4177752

[DEV201259C79] Narendra, V., Rocha, P. P., An, D., Raviram, R., Skok, J. A., Mazzoni, E. O. and Reinberg, D. (2015). CTCF establishes discrete functional chromatin domains at the Hox clusters during differentiation. *Science* 347, 1017-1021. 10.1126/science.1262088 25722416 PMC4428148

[DEV201259C80] Narendra, V., Bulajić, M., Dekker, J., Mazzoni, E. O. and Reinberg, D. (2016). CTCF-mediated topological boundaries during development foster appropriate gene regulation. *Genes Dev.* 30, 2657-2662. 10.1101/gad.288324.116 28087711 PMC5238725

[DEV201259C81] Neijts, R., Amin, S., van Rooijen, C., Tan, S., Creyghton, M. P., de Laat, W. and Deschamps, J. (2016). Polarized regulatory landscape and Wnt responsiveness underlie Hox activation in embryos. *Genes Dev.* 30, 1937-1942. 10.1101/gad.285767.116 27633012 PMC5066237

[DEV201259C82] Niederreither, K. and Dollé, P. (2008). Retinoic acid in development: towards an integrated view. *Nat. Rev. Genet.* 9, 541-553. 10.1038/nrg2340 18542081

[DEV201259C83] Nolte, C., Amores, A., Nagy Kovács, E., Postlethwait, J. and Featherstone, M. (2003). The role of a retinoic acid response element in establishing the anterior neural expression border of Hoxd4 transgenes. *Mech. Dev.* 120, 325-335. 10.1016/S0925-4773(02)00442-2 12591602

[DEV201259C84] Nolte, C., Jinks, T., Wang, X., Martinez Pastor, M. T. and Krumlauf, R. (2013). Shadow enhancers flanking the HoxB cluster direct dynamic Hox expression in early heart and endoderm development. *Dev. Biol.* 383, 158-173. 10.1016/j.ydbio.2013.09.016 24055171

[DEV201259C85] Nolte, C., De Kumar, B. and Krumlauf, R. (2019). Hox genes: Downstream “effectors” of retinoic acid signaling in vertebrate embryogenesis. *Genesis* 57, e23306. 10.1002/dvg.23306 31111645

[DEV201259C86] Noordermeer, D., Leleu, M., Splinter, E., Rougemont, J., De Laat, W. and Duboule, D. (2011). The dynamic architecture of Hox gene clusters. *Science* 334, 222-225. 10.1126/science.1207194 21998387

[DEV201259C87] Noordermeer, D., Leleu, M., Schorderet, P., Joye, E., Chabaud, F. and Duboule, D. (2014). Temporal dynamics and developmental memory of 3D chromatin architecture at Hox gene loci. *eLife* 3, e02557. 10.7554/eLife.02557 24843030 PMC4017647

[DEV201259C88] Nuckolls, N. L., Mok, A. C., Lange, J. J., Yi, K., Kandola, T. S., Hunn, A. M., McCroskey, S., Snyder, J. L., Bravo Núñez, M. A., McClain, M. et al. (2020). The wtf4 meiotic driver utilizes controlled protein aggregation to generate selective cell death. *eLife* 9, e55694. 10.7554/eLife.55694 33108274 PMC7591262

[DEV201259C89] Oosterveen, T., Niederreither, K., Dolle, P., Chambon, P., Meijlink, F. and Deschamps, J. (2003a). Retinoids regulate the anterior expression boundaries of 5′ Hoxb genes in posterior hindbrain. *EMBO J.* 22, 262-269. 10.1093/emboj/cdg029 12514132 PMC140104

[DEV201259C90] Oosterveen, T., van Vliet, P., Deschamps, J. and Meijlink, F. (2003b). The direct context of a hox retinoic acid response element is crucial for its activity. *J. Biol. Chem.* 278, 24103-24107. 10.1074/jbc.M300774200 12697756

[DEV201259C91] Paris, M., Kaplan, T., Li, X. Y., Villalta, J. E., Lott, S. E. and Eisen, M. B. (2013). Extensive divergence of transcription factor binding in Drosophila embryos with highly conserved gene expression. *PLoS Genet.* 9, e1003748. 10.1371/journal.pgen.1003748 24068946 PMC3772039

[DEV201259C92] Parker, H. J. and Krumlauf, R. (2017). Segmental arithmetic: summing up the *Hox* gene regulatory network for hindbrain development in chordates. *Wiley Interdiscip. Rev. Dev. Biol.* 6, e286. 10.1002/wdev.286 28771970

[DEV201259C93] Parker, H. J., De Kumar, B., Green, S. A., Prummel, K. D., Hess, C., Kaufman, C. K., Mosimann, C., Wiedemann, L. M., Bronner, M. E. and Krumlauf, R. (2019). A Hox-TALE regulatory circuit for neural crest patterning is conserved across vertebrates. *Nat. Commun.* 10, 1189. 10.1038/s41467-019-09197-8 30867425 PMC6416258

[DEV201259C94] Philippidou, P. and Dasen, J. S. (2013). Hox genes: choreographers in neural development, architects of circuit organization. *Neuron* 80, 12-34. 10.1016/j.neuron.2013.09.020 24094100 PMC3835187

[DEV201259C95] Pownall, M. E., Tucker, A. S., Slack, J. M. W. and Isaacs, H. V. (1996). eFGF, *Xcad3* and *Hox* genes form a molecular pathway that establishes the anteroposterior axis in *Xenopus*. *Development* 122, 3881-3892. 10.1242/dev.122.12.3881 9012508

[DEV201259C96] Pultz, M. A., Diederich, R. J., Cribbs, D. L. and Kaufman, T. C. (1988). The proboscipedia locus of the Antennapedia complex: a molecular and genetic analysis. *Genes Dev.* 2, 901-920. 10.1101/gad.2.7.901 2850265

[DEV201259C97] Qian, P., De Kumar, B., He, X. C., Nolte, C., Gogol, M., Ahn, Y., Chen, S., Li, Z., Xu, H., Perry, J. M. et al. (2018). Retinoid-sensitive epigenetic regulation of the Hoxb cluster maintains normal hematopoiesis and inhibits leukemogenesis. *Cell Stem Cell* 22, 740-754.e7. 10.1016/j.stem.2018.04.012 29727682

[DEV201259C98] Quinonez, S. C. and Innis, J. W. (2014). Human HOX gene disorders. *Mol. Genet. Metab.* 111, 4-15. 10.1016/j.ymgme.2013.10.012 24239177

[DEV201259C99] Raj, A. and Tyagi, S. (2010). Detection of individual endogenous RNA transcripts in situ using multiple singly labeled probes. *Methods Enzymol.* 472, 365-386. 10.1016/S0076-6879(10)72004-8 20580972

[DEV201259C100] Rhinn, M. and Dollé, P. (2012). Retinoic acid signalling during development. *Development* 139, 843-858. 10.1242/dev.065938 22318625

[DEV201259C101] Rodríguez-Carballo, E., Lopez-Delisle, L., Yakushiji-Kaminatsui, N., Ullate-Agote, A. and Duboule, D. (2019). Impact of genome architecture on the functional activation and repression of Hox regulatory landscapes. *BMC Biol.* 17, 55. 10.1186/s12915-019-0677-x 31299961 PMC6626364

[DEV201259C102] Ronneberger, O., Fischer, P. and Brox, T. (2015). U-Net: Convolutional Networks for Biomedical Image Segmentation. *arXiv*. 10.48550/arXiv.1505.04597

[DEV201259C103] Sanyal, A., Lajoie, B. R., Jain, G. and Dekker, J. (2012). The long-range interaction landscape of gene promoters. *Nature* 489, 109-113. 10.1038/nature11279 22955621 PMC3555147

[DEV201259C104] Schilling, T. F., Nie, Q. and Lander, A. D. (2012). Dynamics and precision in retinoic acid morphogen gradients. *Curr. Opin. Genet. Dev.* 22, 562-569. 10.1016/j.gde.2012.11.012 23266215 PMC3790664

[DEV201259C105] Schoenfelder, S. and Fraser, P. (2019). Long-range enhancer-promoter contacts in gene expression control. *Nat. Rev. Genet.* 20, 437-455. 10.1038/s41576-019-0128-0 31086298

[DEV201259C106] Scotti, M. and Kmita, M. (2012). Recruitment of 5′ Hoxa genes in the allantois is essential for proper extra-embryonic function in placental mammals. *Development* 139, 731-739. 10.1242/dev.075408 22219351 PMC4508127

[DEV201259C107] Serano, J. M., Martin, A., Liubicich, D. M., Jarvis, E., Bruce, H. S., La, K., Browne, W. E., Grimwood, J. and Patel, N. H. (2016). Comprehensive analysis of Hox gene expression in the amphipod crustacean Parhyale hawaiensis. *Dev. Biol.* 409, 297-309. 10.1016/j.ydbio.2015.10.029 26569556

[DEV201259C108] Sharpe, J., Nonchev, S., Gould, A., Whiting, J. and Krumlauf, R. (1998). Selectivity, sharing and competitive interactions in the regulation of *Hoxb* genes. *EMBO J.* 17, 1788-1798. 10.1093/emboj/17.6.1788 9501100 PMC1170526

[DEV201259C109] Simeone, A., Acampora, D., Arcioni, L., Andrews, P. W., Boncinelli, E. and Mavilio, F. (1990). Sequential activation of HOX2 homeobox genes by retinoic acid in human embryonal carcinoma cells. *Nature* 346, 763-766. 10.1038/346763a0 1975088

[DEV201259C110] Sirbu, I. O., Gresh, L., Barra, J. and Duester, G. (2005). Shifting boundaries of retinoic acid activity control hindbrain segmental gene expression. *Development* 132, 2611-2622. 10.1242/dev.01845 15872003 PMC2833012

[DEV201259C111] Soshnikova, N. and Duboule, D. (2009). Epigenetic temporal control of mouse Hox genes in vivo. *Science* 324, 1320-1323. 10.1126/science.1171468 19498168

[DEV201259C112] Studer, M., Pöpperl, H., Marshall, H., Kuroiwa, A. and Krumlauf, R. (1994). Role of a conserved retinoic acid response element in rhombomere restriction of *Hoxb-*1. *Science* 265, 1728-1732. 10.1126/science.7916164 7916164

[DEV201259C113] Sucov, H. M., Murakami, K. K. and Evans, R. M. (1990). Characterization of an autoregulated response element in the mouse retinoic acid receptor type beta gene. *Proc. Natl. Acad. Sci. USA* 87, 5392-5396. 10.1073/pnas.87.14.5392 2164682 PMC54330

[DEV201259C114] Tarchini, B. and Duboule, D. (2006). Control of Hoxd genes’ collinearity during early limb development. *Dev. Cell* 10, 93-103. 10.1016/j.devcel.2005.11.014 16399081

[DEV201259C115] Tsai, A., Muthusamy, A. K., Alves, M. R. P., Lavis, L. D., Singer, R. H., Stern, D. L. and Crocker, J. (2017). Nuclear microenvironments modulate transcription from low-affinity enhancers. *eLife* 6, e28975. 10.7554/eLife.28975 29095143 PMC5695909

[DEV201259C116] Tsai, A., Alves, M. R. P. and Crocker, J. (2019). Multi-enhancer transcriptional hubs confer phenotypic robustness. *eLife* 8, e45325. 10.7554/eLife.45325 31294690 PMC6650246

[DEV201259C117] Tschopp, P., Christen, A. J. and Duboule, D. (2012). Bimodal control of Hoxd gene transcription in the spinal cord defines two regulatory subclusters. *Development* 139, 929-939. 10.1242/dev.076794 22278926

[DEV201259C118] Tümpel, S., Maconochie, M., Wiedemann, L. M. and Krumlauf, R. (2002). Conservation and diversity in the cis-regulatory networks that integrate information controlling expression of Hoxa2 in hindbrain and cranial neural crest cells in vertebrates. *Dev. Biol.* 246, 45-56. 10.1006/dbio.2002.0665 12027433

[DEV201259C119] Umesono, K., Murakami, K. K., Thompson, C. C. and Evans, R. M. (1991). Direct repeats as selective response elements for the thyroid hormone, retinoic acid, and vitamin D3 receptors. *Cell* 65, 1255-1266. 10.1016/0092-8674(91)90020-Y 1648450 PMC6159884

[DEV201259C120] Zaffran, S. and Kelly, R. G. (2012). New developments in the second heart field. *Differentiation* 84, 17-24. 10.1016/j.diff.2012.03.003 22521611

[DEV201259C121] Zeitlinger, J. (2020). Seven myths of how transcription factors read the cis-regulatory code. *Curr. Opin. Syst. Biol.* 23, 22-31. 10.1016/j.coisb.2020.08.002 33134611 PMC7592701

[DEV201259C122] Zhang, Z. and Tjian, R. (2018). Measuring dynamics of eukaryotic transcription initiation: Challenges, insights and opportunities. *Transcription* 9, 159-165. 10.1080/21541264.2017.1363017 28920762 PMC5927711

[DEV201259C123] Zuin, J., Roth, G., Zhan, Y., Cramard, J., Redolfi, J., Piskadlo, E., Mach, P., Kryzhanovska, M., Tihanyi, G., Kohler, H. et al. (2022). Nonlinear control of transcription through enhancer-promoter interactions. *Nature* 604, 571-577. 10.1038/s41586-022-04570-y 35418676 PMC9021019

