# Supplementary material for: Shared retinoic acid responsive enhancers coordinately regulate nascent transcription of Hoxb coding and non-coding RNAs in the developing mouse neural tube
Source: Development. 2023 May 24;150(10):dev201259. doi: 10.1242/dev.201259 (PMC10233718; doi:10.1242/dev.201259)
Supplement: Supplementary information [file develop-150-201259-s1.pdf]

Fig. S1

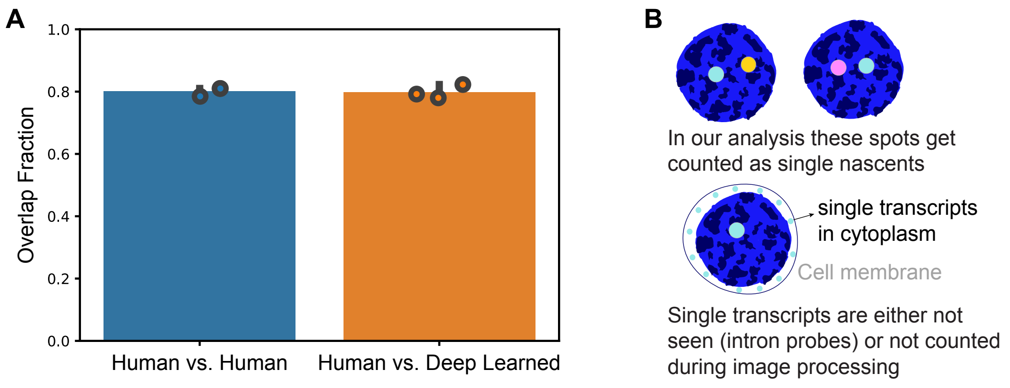

Fig. S1. Reproducibility in calling spots of nascent transcripts. A Comparing accuracy of DL against manual markings of spots. **B** Cell schematics to indicate what doesn't get counted as co-localized using Deep learning and a diagram to show that single transcripts in the cytoplasms do not counted.

Development • Supplementary information

Fig. S2

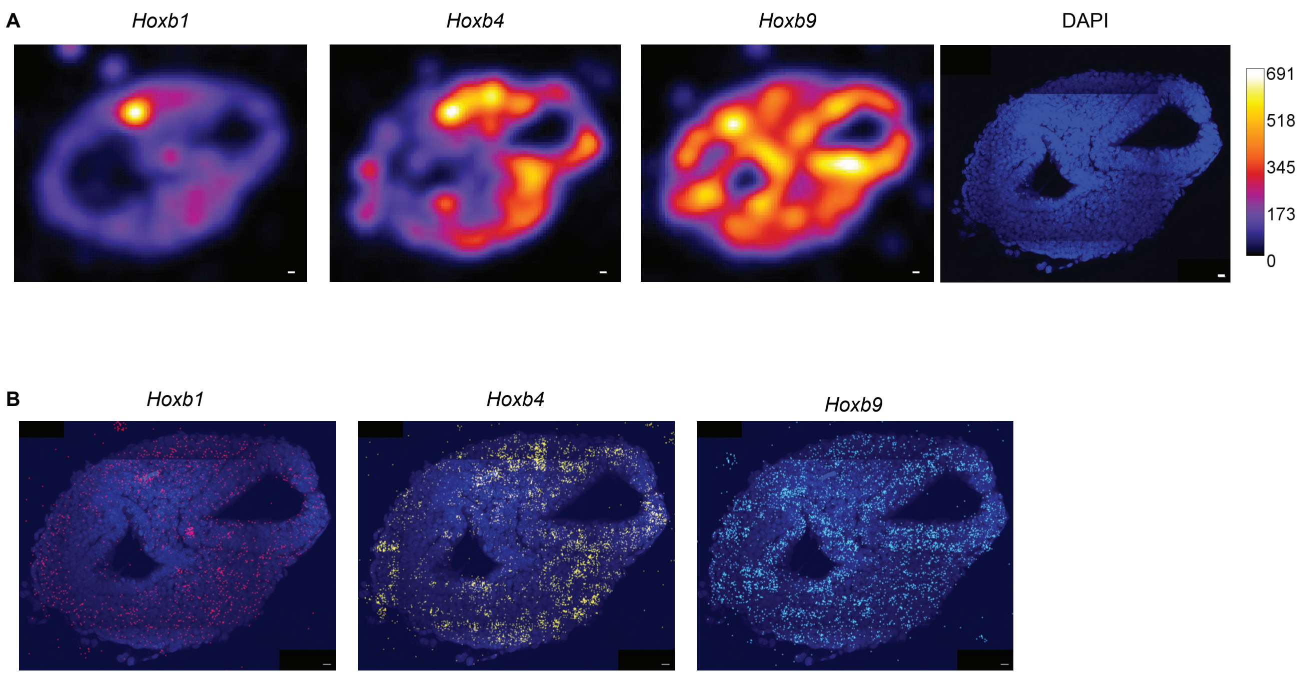

Fig. S2. Intensity plots and spot fitting of nascent transcripts overlapping with DAPI staining.

A Intensity plots to visualize regions of high or low nascent transcripts using fire LUT; white/yellow indicate higher numbers of nascent transcripts while blue/purple indicates lower numbers of nascent transcripts. **B** Spot fitting algorithm to visualize nascent transcripts overlapping with DAPI stained nuclei in the mouse tail.

Development • Supplementary information

Fig. S3

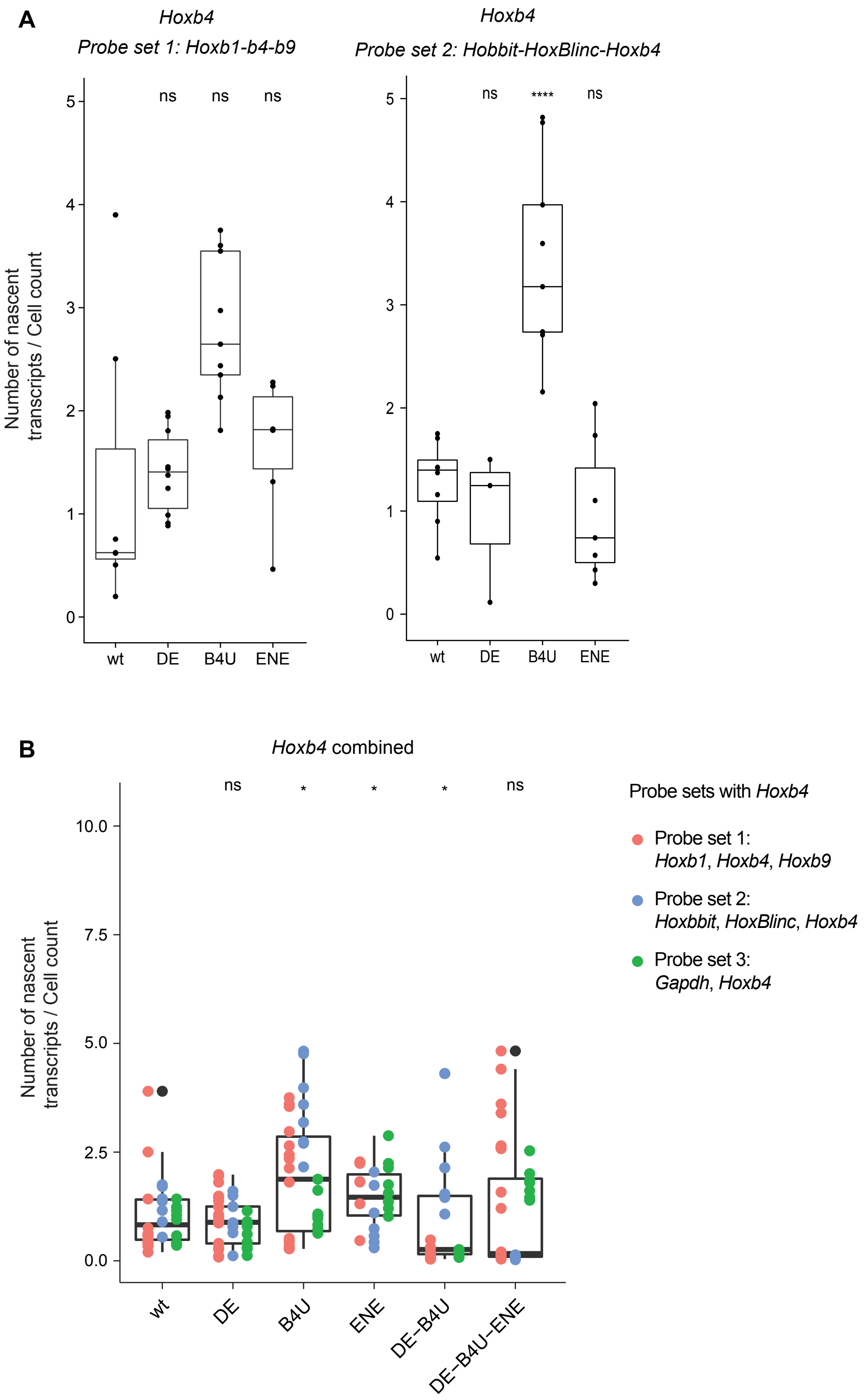

Fig. S3. Quantification of total number of *Hoxb4* nascent transcripts/cell in wild type and mutant embryos calculated as an average of data from multiple near adjacent tail sections. A Box plot of average levels of nascent *Hoxb4* expression measured using with two different probe sets on a series alternate tissue sections from single embryo of wild type and mutant mouse lines. **B** Box plot of average levels of nascent *Hoxb4* expression calculated by combining data from three different probe sets and multiple embryos. Each color represents a different probe set. Probes for the genes in each set are indicated at the right.

Development • Supplementary information

Fig. S4

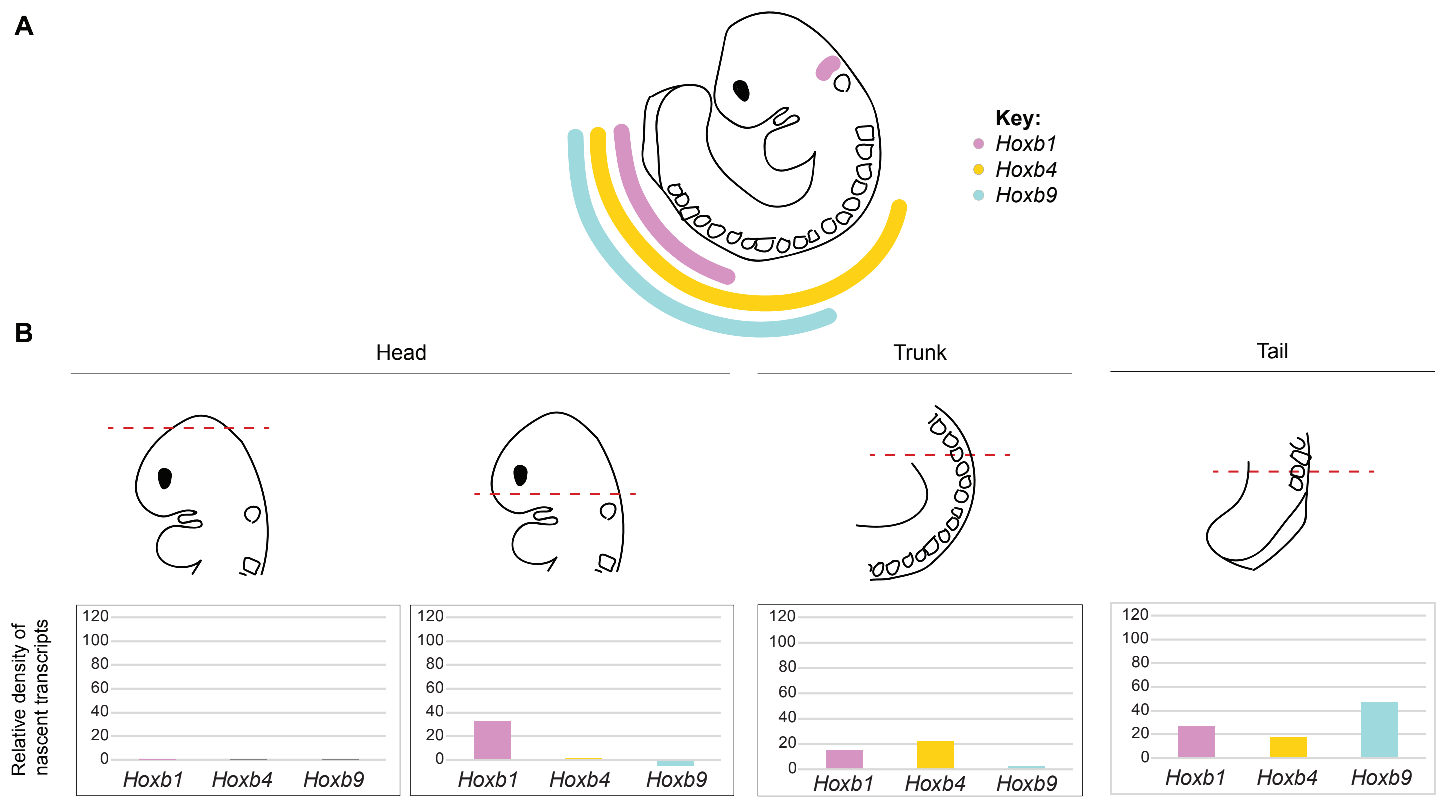

Fig. S4. Comparison of detected *Hoxb n*ascent transcripts against known expression. A Schematic illustrating known A-P expression patterns for steady state levels of *Hoxb1*, *Hoxb4*, and *Hoxb9* coding genes detected by conventional colorimetric in situs in a 9.5 dpc mouse embryo. **B** At the top, a schematic of transverse sections (indicated by dotted line) at different A-P levels through neural tube of a 9.5 dpc embryo. Below each section are representative bar graphs indicating the relative densities in patterns of nascent transcription for *Hoxb1*, *Hoxb4* and *Hoxb9*. The shifts in level along the A-P axis mirror those illustrated in panel **A,** with *Hoxb4* and *Hoxb9* missing in anterior regions and elevated in posterior regions.

Development • Supplementary information

Fig. S5

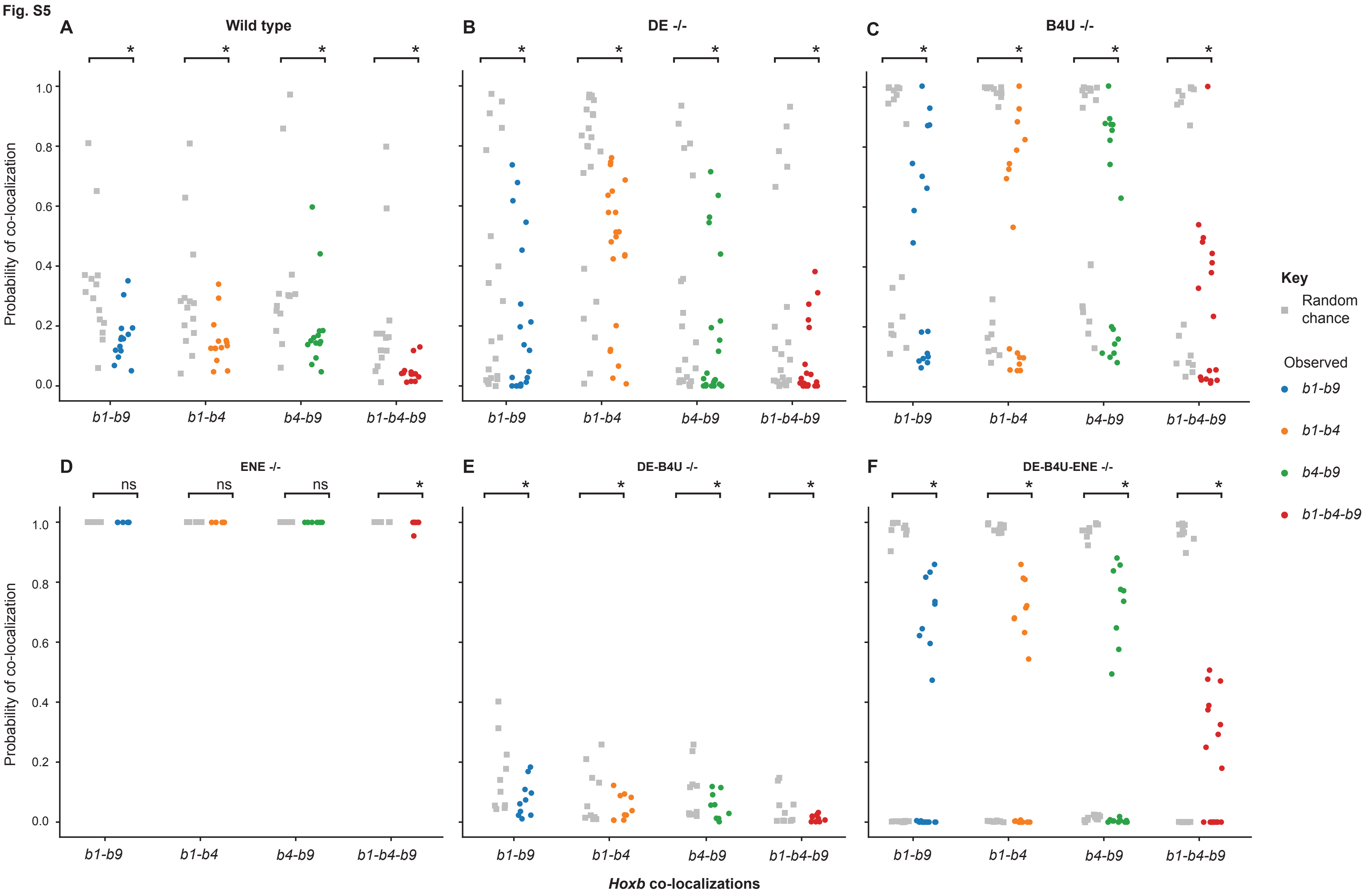

Fig. S5. Observed and random probabilities for co-localization of Hoxb nascent transcripts. A-F Probabilities of co-localization by random chance (grey) next to observed co-localization probabilities (colored) for coding *Hoxb* genes in wild type and RARE mutants. Random chance of co-localization was calculated based on the numbers of single transcripts expressed within a defined area. The observed values are compared against what would occur by random chance in single and compound RARE mutants, and significant difference between the values is calculated by Wilcoxon signed-rank test and denoted by asterisks.

Fig. S6

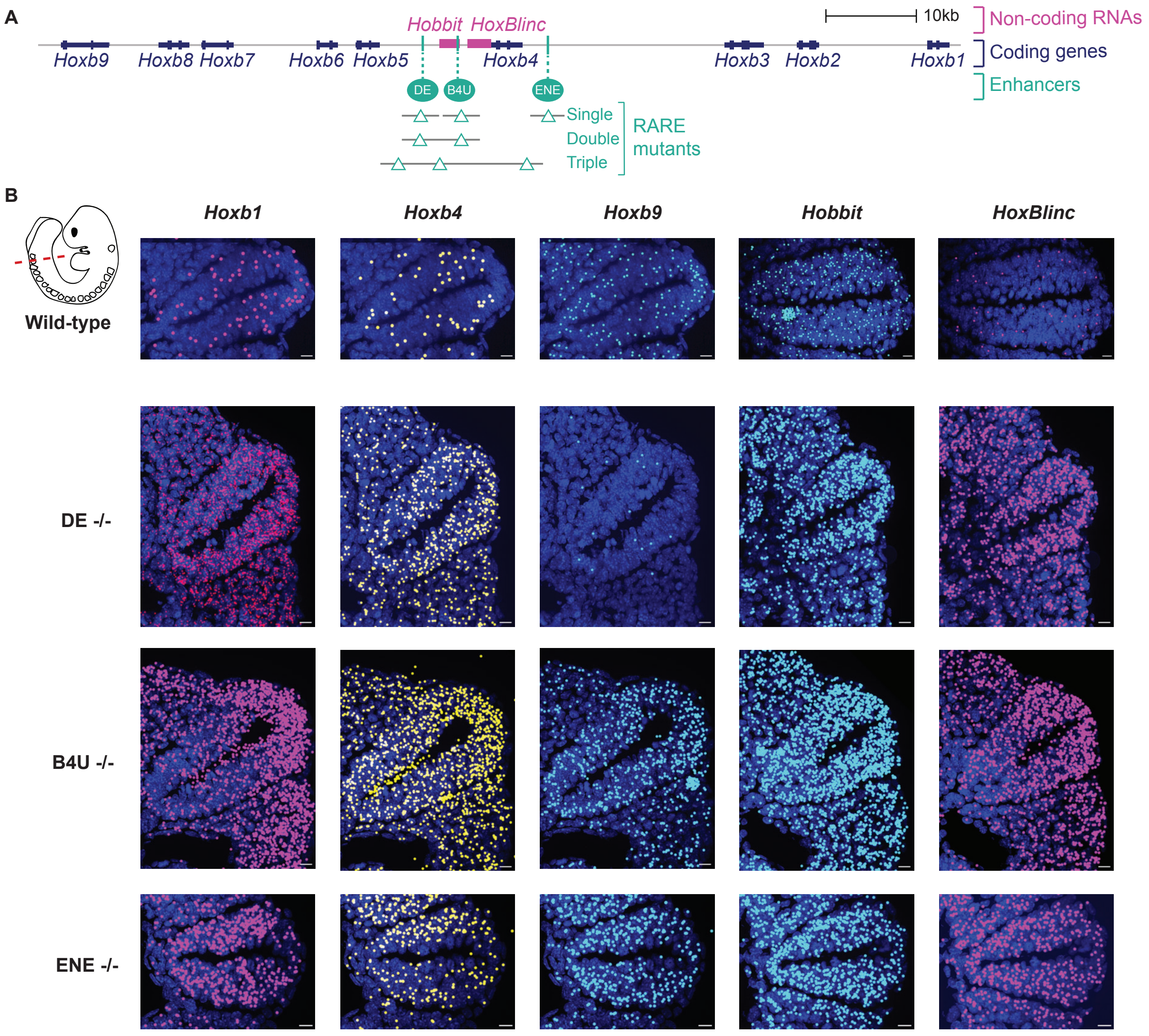

Fig. S6. Visualizing changes in levels of nascent transcription of Hoxb coding and non-coding genes in single mutants of three different shared RARE enhancers.

A Diagram depicting a series of single and compound mutants generated in RAREs of three shared enhancers present in the center of the *Hoxb* cluster. **B** Images of nascent transcripts for *Hoxb* coding and non-coding genes in wild type and the series of single RARE mutant embryos. The nascent transcripts detected by DL were spot fitted to increase the size of the spots for better visualization over the neural tube.

Development • Supplementary information

Fig. S7

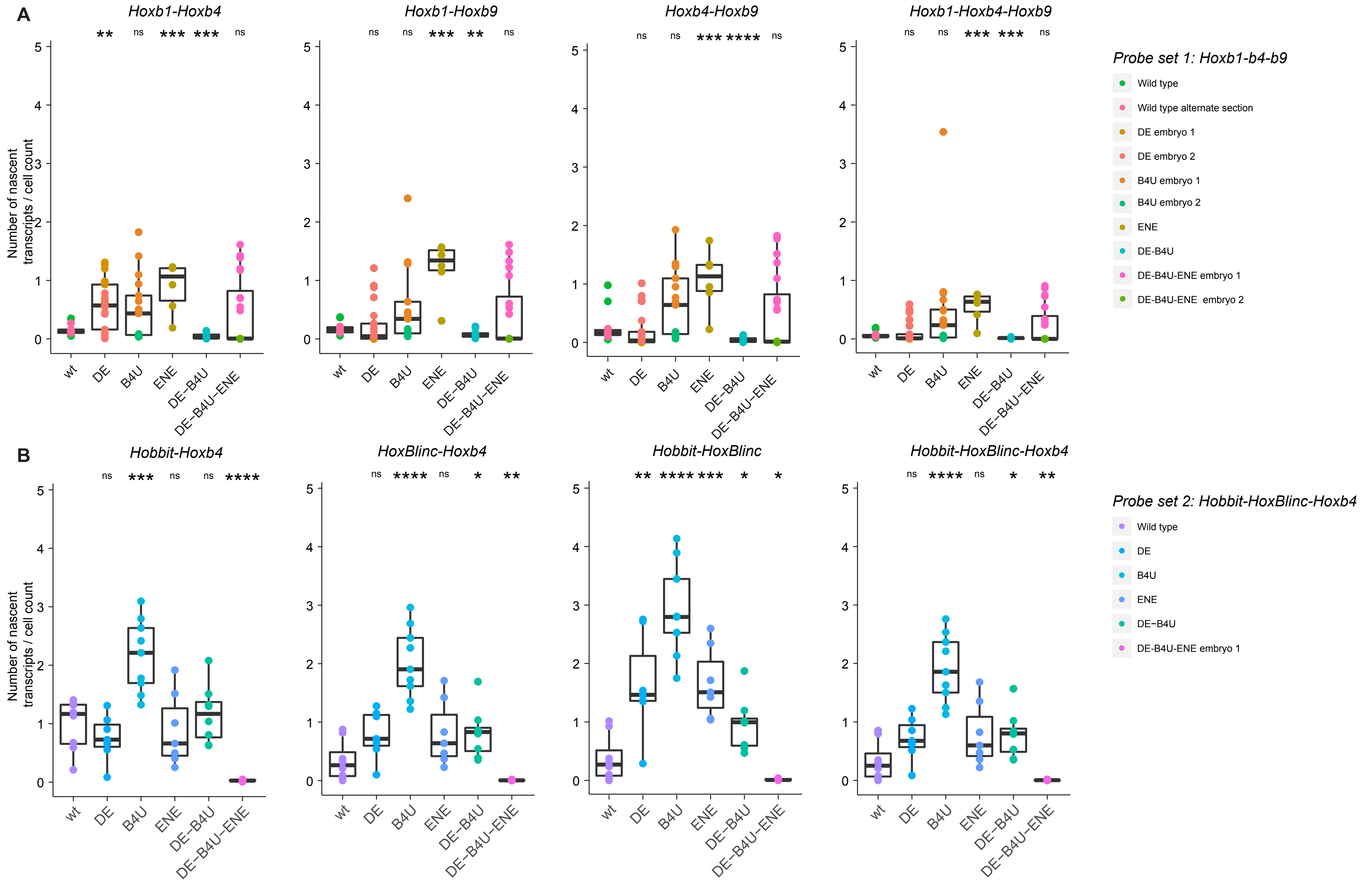

Fig. S7. Quantifying patterns of co-localization of nascent transcription in wild type and mutant embryos.
Box plots show the number of co-localized double and triple combinations of nascent transcripts/cell for *Hoxb* the coding (**A**) and non-coding (**B**) genes. Values are calculated as an average of combined data from multiple near adjacent tail sections (7-9) of multiple embryos. Each box in A and B represents the 75th (top line) and 25th (bottom line) percentile while the middle line represents the median expression.

Development • Supplementary information

Fig. S8

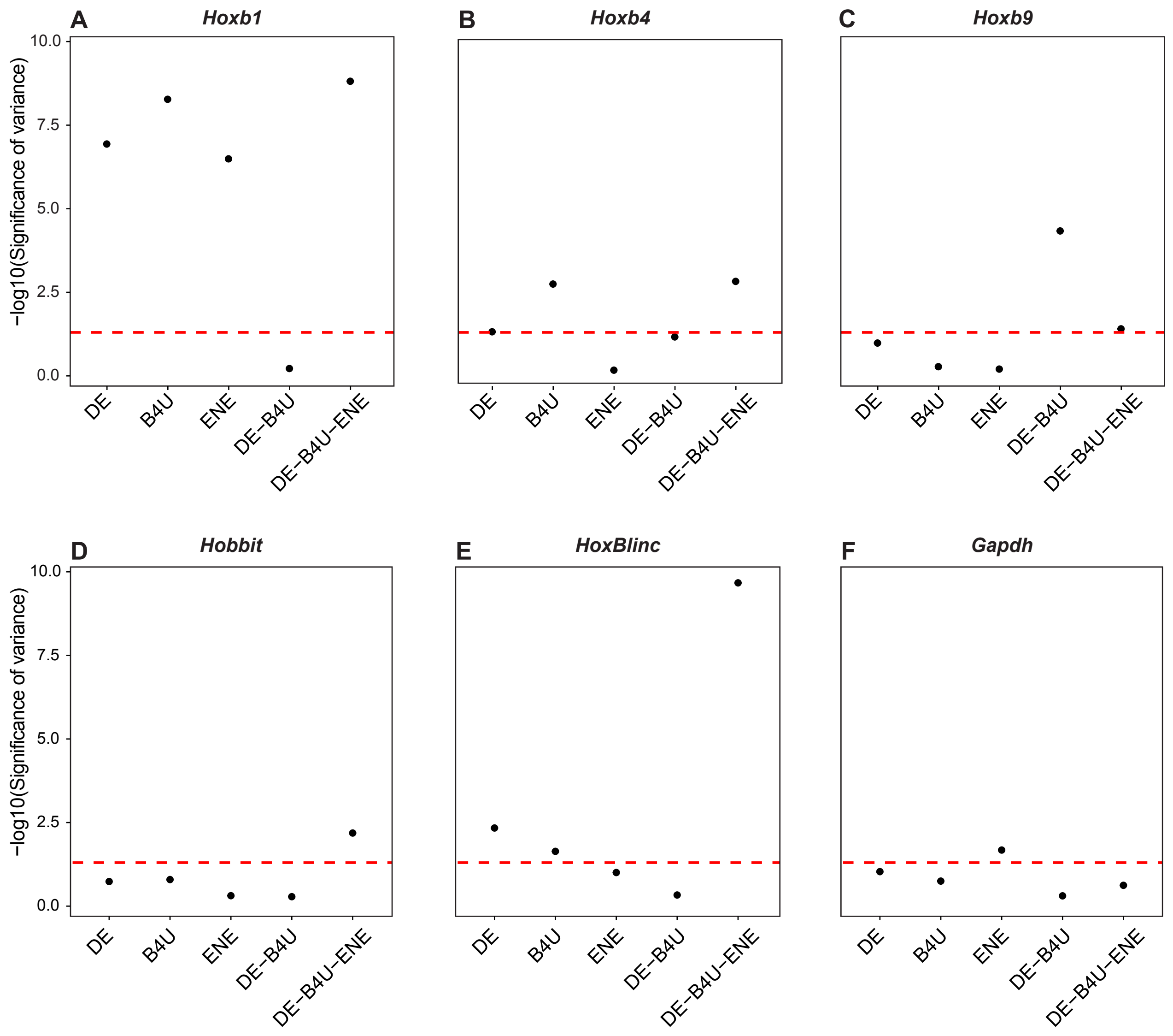

Fig. S8. Variance plots for the difference in nascent transcripts in RARE mutants compared to wild type embryos. A-F Dot plots representing significance of variance in expression of *Hoxb* transcripts in RARE mutants compared to wild type. Red line represents p-value of 0.05 and thus any dot above the line is statistically significant for increased variance in expression of nascent transcripts among tissue sections compared to wild type.

Table S1. List of smFISH probes and their corresponding fluorophore.
Three probe set combinations were used so genes could be imaged simultaneously without needing to spectrally unmix signals. Probe set 1 combined: *Hoxb9*-647, *Hoxb1*-570, and *Hoxb4*- 488, Probe set 2: *Hobbit*-647, *HoxBlinc*-555, and *Hoxb4*-488, and Probe set 3: *Gapdh*-670 and *Hoxb4*-488.

Genes	Labelled with fluorophore
Hoxb1 intron spanning (intron and exon)	Quasar 570
Hoxb4 intron spanning (intron and exon)	Atto 488
Hoxb4 intron	AF 647
Hoxb9 intron	AF 647
Hobbit	AF 647
HoxBlinc	AF 555
Gapdh exon	Quasar 670

Development • Supplementary information

Table S2. List of exact p-values calculated for Fig S5. The observed values are compared against what would occur by random chance in wild-type, single and compound RARE mutants, and significant difference between the values is calculated by Wilcoxon signed-rank test. In Fig S5 if p-value is less than 0.05, it is denoted by asterisks.

Sample	Chanel of co-localized spots	P-value	P- value <0.05
Wild-type	Hoxb1 and Hoxb4	0.00024414	TRUE
Wild-type	Hoxb1 and Hoxb9	0.00012207	TRUE
Wild-type	Hoxb4 and Hoxb9	0.00012207	TRUE
Wild-type	Hoxb1, Hoxb4, and Hoxb9	0.00012207	TRUE
DE -/-	Hoxb1 and Hoxb4	9.54E-07	TRUE
DE -/-	Hoxb1 and Hoxb9	0.00010335	TRUE
DE -/-	Hoxb4 and Hoxb9	0.00010335	TRUE
DE -/-	Hoxb1, Hoxb4, and Hoxb9	0.00010335	TRUE
B4U -/-	Hoxb1 and Hoxb4	1.53E-05	TRUE
B4U -/-	Hoxb1 and Hoxb9	1.53E-05	TRUE
B4U -/-	Hoxb4 and Hoxb9	1.53E-05	TRUE
B4U -/-	Hoxb1, Hoxb4, and Hoxb9	1.53E-05	TRUE
ENE -/-	Hoxb1 and Hoxb4	0.17971249	FALSE
ENE -/-	Hoxb1 and Hoxb9	0.31731051	FALSE
ENE -/-	Hoxb4 and Hoxb9	0.31731051	FALSE
ENE -/-	Hoxb1, Hoxb4, and Hoxb9	0.03125	TRUE
DE-B4U -/-	Hoxb1 and Hoxb4	0.03710938	TRUE
DE-B4U -/-	Hoxb1 and Hoxb9	0.00195313	TRUE
DE-B4U -/-	Hoxb4 and Hoxb9	0.00195313	TRUE
DE-B4U -/-	Hoxb1, Hoxb4, and Hoxb9	0.00976563	TRUE
DE-B4U-ENE -/-	Hoxb1 and Hoxb4	0.00053852	TRUE
DE-B4U-ENE -/-	Hoxb1 and Hoxb9	0.00039814	TRUE
DE-B4U-ENE -/-	Hoxb4 and Hoxb9	0.00013183	TRUE
DE-B4U-ENE -/-	Hoxb1, Hoxb4, and Hoxb9	0.00013183	TRUE

Table S3. The specific mutations generated in the endogenous *DE*, *B4U* and *ENE* *RAREs* of the *Hoxb* cluster. The mutated sequences represent base pair substitutions (indicated in red) that disrupt RAR/RXR binding sites but maintain the same spatial distances between these and other cis-elements in the endogenous genomic locus. The sequences of the direct repeats (DR) in these RAREs are indicated in capital letters.

**RARE**	***DE*** (DR5)	***B4U*** (DR5)	***ENE*** (DR5)
**Wildtype sequence**	GGATCAcgcagAGGTCA	GGGTGAaccgcAGGTCA	AGTTCAtggagAGGCCA
**Mutated sequence**	GG**CC**CAcgcagA**CAA**CA	G**AATTC**acc**agTTC**TCA	A**CCAAG**tggac**T**G**AATT**

Table S4. List of specific CRISPR guide and Homology Directed Repair (HDR) templates used to generate the *DE*, *B4U* and *ENE* RARE mutants in the endogenous *Hoxb* cluster. The sequences of CRISPR guides were ordered as oligos and the HDR templates were ordered as ultramers (IDT) and used to generate the RARE mutants in the Stowers F1 mouse strains. The *DE-B4U* double mutant was generated by microinjections of *B4U* guides in *DE-RARE* mutant animals, and the *DE-B4U-ENE* triple mutant was generated by microinjections of *ENE* guides in *DE-B4U RARE* mutant animals.

	**CRISPR 5' Guide**	**HDR template**
DE	Previously described in Ahn et.al *Development* 2014.	
B4U	5'-GAGGGGTGAACCGCAGGTCA- 3'	5'CGAGCGAACTGCGTGAGCATATTATACTAACT GCCTGCTCGTGGGGGAGGCCCGGAGAGGAATT CACCAGTTCTCACGGCGTCTAAAAATTATTAAAA TGTTCGAGAACCTCGTGACGCGCCTGCTGTTTA ACAAAG 3'
ENE	5'-GAGGAGGAAGAGTTCATGGAG-3'	5'CCTTTCCCTGAGGCCTCACTGAGAGAGAGTTT GGAGGCGGGAGGATGGCAGGAGGAAGACCAAG TGGACTGAATTCGTTTTCTAGGCGATTAGCTTGG TATAGAACCACAAAATGACCTCGGAAGCCCCTT GACCTCTC 3'

Development • Supplementary information
