# Supplementary material for: Shared retinoic acid responsive enhancers coordinately regulate nascent transcription of Hoxb coding and non-coding RNAs in the developing mouse neural tube
Source: Development. 2023 May 24;150(10):dev201259. doi: 10.1242/dev.201259 (PMC10233718; doi:10.1242/dev.201259)
Supplement: Supplementary file 1 [file develop-150-201259_review_history.pdf]

Shared retinoic acid responsive enhancers coordinately regulate nascent transcription of *Hoxb* coding and non-coding RNAs in the developing mouse neural tube

Zainab Afzal, Jeffrey Lange, Christof Nolte, Sean McKinney, Christopher Wood, Ariel Paulson, Bony De Kumar, Jay Unruh, Brian D. Slaughter and Robb Krumlauf
DOI: 10.1242/dev.201259

Editor: Debra Silver

Review timeline

Original submission:	1 September 2022
Editorial decision:	8 November 2022
First revision received:	22 February 2023
Editorial decision:	11 April 2023
Second revision received:	17 April 2023
Accepted:	19 April 2023

Original submission

First decision letter

MS ID#: DEVELOP/2022/201259

MS TITLE: Shared retinoic acid responsive enhancers coordinately regulate nascent transcription of Hoxb coding and non-coding RNAs in the developing mouse neural tube

AUTHORS: Zainab Afzal, Jeffrey Lange, Christof Nolte, Sean McKinney, Christopher Wood, Ariel Paulson, Bony De Kumar, Jay Unruh, Brian D Slaughter, and Robb Krumlauf

I have now received all the referees' reports on the above manuscript, and have reached a decision. The referees' comments are appended below, or you can access them online: please go to BenchPress and click on the 'Manuscripts with Decisions' queue in the Author Area.

As you will see, the referees express considerable interest in your work, but have some significant criticisms and recommend a revision of your manuscript before we can consider publication. Specifically, reviewer 1 suggests some discussion regarding your findings relative to 3D organization. Both reviewers 2 and 3 suggest the need for more robust statistical analyses and also have several suggestions for clarity to make the paper easier to follow. If you are able to revise the manuscript along the lines suggested, which may involve further experiments, I will be happy receive a revised version of the manuscript. Your revised paper will be re-reviewed by one or more of the original referees, and acceptance of your manuscript will depend on your addressing satisfactorily the reviewers' major concerns. Please also note that Development will normally permit only one round of major revision. If it would be helpful, you are welcome to contact us to discuss your revision in greater detail. Please send us a point-by-point response indicating your plans for addressing the referee’s comments, and we will look over this and provide further guidance.

Please attend to all of the reviewers' comments and ensure that you clearly highlight all changes made in the revised manuscript. Please avoid using 'Tracked changes' in Word files as these are lost in PDF conversion. I should be grateful if you would also provide a point-by-point response detailing

© 2023. Published by The Company of Biologists under the terms of the Creative Commons Attribution License (https://creativecommons.org/licenses/by/4.0/).

how you have dealt with the points raised by the reviewers in the 'Response to Reviewers' box. If you do not agree with any of their criticisms or suggestions please explain clearly why this is so.

Reviewer 1

Advance summary and potential significance to field

Afzal et al. present an in-depth, quantitative analysis of Hoxb regulation in the neural tubes of mouse embryos. The experiments are nicely executed and the analysis is thoughtful. The authors present evidence that three linked RA-dependent enhancers interact to control precise patterns and levels of Hoxb transcription. Deletions of individual and combinations of enhancers cause complex changes in transcription. For example, deletion of the B4U enhancer leads to increases in nearby transcription units, while the double deletion (DE-B4U) restores nearly normal levels of expression. The authors propose that these changes in expression could be explained by the association of the three enhancers within a common regulatory hub.

Comments for the author

If possible, it would be nice to discuss some of the findings in the context of the 3D organization of the Hoxb locus. Are previously published Hi-C maps consistent with a common regulatory hub containing the three RA enhancers?

Reviewer 2

Advance summary and potential significance to field

In this manuscript from Afzal et al., the authors use single-cell, single-molecule approaches for interrogating the cis-regulatory logic of Hoxb genes in mouse embryos. It's an inspired choice for this type of analysis: the Hoxb locus has a complicated topology and is undoubtedly important for vertebrate development. The primary finding is that there is no evidence for co-transcriptional coupling of genes in the cluster. The authors then do a deep dive with mutagenesis of the various regulatory elements. Overall, I found the approach to be well-suited to the questions, but the narrative was hard to follow at times. There is a large body of work in the fly blastoderm on similar questions but little if any (to my knowledge) on vertebrates, let alone mouse. In that sense, this work is a technical advance. I think some revisions are required, but it seems like a strong fit for Development.

Comments for the author

Major comments:
1. There seems to be a substantial amount of background in the intronic signal which is usually very clean necessitating a Deep Learning approach for transcription site segmentation. I don't think there is much to compare to when it comes to smFISH in mouse embryos, but the amount of fluorescence background diminishes by enthusiasm somewhat.
In addition, the co-localiztion distance of 1.5 um is rather large and raises eyebrows. Do any results change substantially with a cutoff of < 1 um? A false positive rate of 20% (taken from the Gapdh-Hoxb4 probeset) is an appreciable error.

2. There needs to be a more rigorous statistical treatment and comparison to some sort of null hypothesis for co-transcriptional bursting. The statistical treatment might consist of a Fisher's exact test. The null hypothesis is more difficult: what is the random expectation for co-occurring transcription sites? Are the genes just independent (unaware of each other) or actually anti-correlated? The latter suggests an active regulatory process at the level of individual bursts. I don't think fig. S7 satisfies this requirement. The authors might take a look at a recent paper on coordinated transcription placed on biorxiv (https://doi.org/10.1101/2022.07.07.499202). At present, this conclusion is interesting but not quite substantiated: "This suggests the presence of layers of regulation and enhancer interactions governing patterns of nascent Hoxb expression may include some that restrict transcriptional co-activation."

© 2023. Published by The Company of Biologists under the terms of the Creative Commons Attribution License (https://creativecommons.org/licenses/by/4.0/).

3. Figure 6 description probably needs to be completely re-written. The text is so spare as to be almost uninformative and seems like it was written in haste -- I read this part again and again and still didn't quite get it. One is not directly visualizing the enhancer, correct? These are only nascent RNAs made from transcription units. So what does this subsection title mean: "Enhancer-promoter distances decrease in the RARE mutants"? I suppose the conclusion is that deletion of an enhancer changes the spatial configuration of the locus (Fig. 6C), but that is a different conclusion. Is the plot in Fig. 6D showing that in the ENE-/- mutant the whole locus is more condensed? i.e. area in the yellow triangle is smaller? This experiment seems like an immense amount of work, and maybe the conclusions are profound, but the interpretation is lacking for this referee.

Minor comment:
1. I find it stylistically jarring to go back and forth between Figs. 1 and 2 out of order in the first part of the manuscript.
2. "A caveat of our optimized smFISH protocol is that we could not distinguish single transcripts." If you can't see single transcripts, is it really smFISH?
3. Fig. 6 needs some sort of treatment of statistical significance.

Reviewer 3

Advance summary and potential significance to field

In this manuscript, Afzal and colleagues set out to decouple the mechanisms that regulate the expression of genes and non-coding RNAs within the Hoxb gene cluster. The authors focus on three shared retinoic acid response elements
(RARE) enhancers, and develop an optimized smFISH protocol coupled with deep learning to measure nascent transcription for a subset of genes within the Hoxb cluster in mouse embryonic tissues. The authors report that nascent transcription tends to happen for a single Hoxb gene (of the 6 total elements analyzed) in individual cells at a given time. Single and a combination of mutations in different RARE enhancers affect both nascent transcription of individual genes and coordinated expression across genes and lnc within the cluster. The authors observe some structure in how different enhancer mutations affect specific patterns, suggesting that there are underlying regulatory mechanisms driving robustness in levels and patterns of Hoxb genes.

Overall, this is a well-designed and timely study, bringing in new experimental and computational techniques to mouse neural tube development. The manuscript is clearly motivated, the text is easy to follow, and the authors have made substantial efforts to perform appropriate controls as they bring in smFISH to this stage of mouse tissue. The results are generally supported by the data, and the authors are generally careful and cautious in their conclusions given their observations.

Yogesh Goyal

Comments for the author

I only have a few comments, which I have provided below for the authors to consider.
I found it a bit hard to go back and forth between Figure 1 and Figure 2 as they are not chronologically stated in the maintext. The authors may consider starting with Figure 2 first, or at least combine early parts of Figure 1 with all of Figure 2 to avoid going back and forth between the two.
I am not sure why the authors were not able to visualize single transcripts for any of the genes tested. While I commend the authors for clearly stating this observation, is this because the expression of mRNA is too high or barely detectable? I do not expect them to perform new experiments for this study as it is way beyond the scope of their work, but the authors may want to discuss in their discussion section if newer brighter clampFISH/HCR techniques could be used to address this in the future (e.g. Choi et al Development 2018, or Dardani et al Nature Methods 2022).
Figure S3B is a bit confusing to me. Not sure if they need to compare sums across gene sets, and what that analysis reveals biologically. If the authors have a compelling reason for this analysis, I am curious to know.

© 2023. Published by The Company of Biologists under the terms of the Creative Commons Attribution License (https://creativecommons.org/licenses/by/4.0/).

Figure 3F, the maximum dot is below 5. The authors should have the y-axis that is not too far from the maximum value. I understand they have it until 10 because they compare it to mutants in future figures, but for this figure, it is only appropriate to have the y-axis scaled such that there is not so much empty space.
Figure S7: I do not see any statistical tests to confirm their observations.
This is necessary for the authors to make the conclusion that co-localization is lower than by random chance.
Figure 4 and 5: the statistical significance with “*” and stars are not particularly clear. The authors should consider explaining it clearly in figure caption.
Figure 5B: I like their plot, but I want to clarify to make sure I understand it the way the authors meant it: If each color is a different embryo, why are some colors less number than others (e.g. green for bimodal DE is only one dot. Does it mean that other green dots are hidden behind other dots? If so, the authors should add a small jitter to their plots to see all dots.)
Figure 6B: Why are there no error bars on the distance plots?
Yogesh Goyal

First revision

Author response to reviewers' comments

General author response:
We are grateful to the reviewers for their very helpful and constructive comments. In the revised manuscript, we have tried our best to address, clarify or explain all of the specific points that were raised. This involved rearranging figures to improve the flow of the figures with the text; performing new analysis to clarify levels of resolution; rewriting the final results section; and modifying other areas of text related to addressing reviewers comments. We feel this has really enhanced the continuity and clarity of the manuscript and our conclusions, so we are appreciative of the thoughtful feedback provided by the reviewers.
To help assess and follow these revisions and responses, below we provide a detailed point by point list of comments and changes for each of the comments. The reviewers' comments are shown in black text and our responses in blue italic text.

Reviewer 1 *(Advance Summary and Potential Significance to Field)*
Afzal et al. present an in-depth, quantitative analysis of Hoxb regulation in the neural tubes of mouse embryos. The experiments are nicely executed, and the analysis is thoughtful. The authors present evidence that three linked RA-dependent enhancers interact to control precise patterns and levels of Hoxb transcription. Deletions of individual and combinations of enhancers cause complex changes in transcription. For example, deletion of the B4U enhancer leads to increases in nearby transcription units, while the double deletion (DE-B4U) restores nearly normal levels of expression. The authors propose that these changes in expression could be explained by the association of the three enhancers within a common regulatory hub.

Reviewer 1 Comments for the Author*:*
If possible, it would be nice to discuss some of the findings in the context of the 3D organization of the Hoxb locus.

We thank the reviewer for this suggestion which is also related to point 3 of reviewer 2 asking us to revise the section describing changes in spatial organization in RARE mutants. We have rewritten this final section of results which is now entitled: "Mutations in RAREs change the spatial relationships between promoters in the cluster". This section now includes some discussion of our findings in relation to 3D or spatial relationships.

In addition, we have added the following paragraph to the discussion:
"Relevant to the increased levels of nascent transcription observed in single RARE mutants, we also observed changes in the spatial relationships between promoters in the cluster. Hoxb9 nascent spots appear closer to Hoxb1 and Hoxb4 (Fig. 6) and this increased physical proximity correlates with increased patterns of expression. Finding that mutations in the RAREs of shared enhancers

© 2023. Published by The Company of Biologists under the terms of the Creative Commons Attribution License (https://creativecommons.org/licenses/by/4.0/).

may alter the dynamics of regulatory interactions and physical distances between promoters raises These findings raise the possibility that the Hoxb genomic locus may have a 3D architecture which can serve as a transcriptional hub during co-transcriptional activation of multiple Hoxb genes. Current Hi-C data do not provide sufficient resolution to robustly identify sub-TADs or regulatory hubs within the Hoxb TAD in the developing mouse neural tube.
However, the pairwise distances between nascent spots for co-transcriptionally active Hoxb1,

Hoxb4 and Hoxb9 was 350 nm, which is within the physical space (<1100 nm) that can influence interactions between promoters of genes in close proximity when bursting (Bohrer and Larson, 2022) and consistent with models for a transcriptional hub or shared megadalton transcription factory. In a hub, in which the three RARE enhancers could be organized to dynamically compete or synergize with each other in interacting with and activating their target Hoxb gene promoters. This could potentiate the rapid turning on or off of nascent transcription of individual, and sometimes multiple genes, in a controlled manner."

Are previously published Hi-C maps consistent with a common regulatory hub containing the three RA enhancers?

Unfortunately published Hi-C maps and methods do not have sufficient resolution to robustly define sub-TADs or regulatory hubs, particularly for cases like the Hoxb cluster, which is only 90 kb, and much smaller than megabase sized TADs commonly detected in the genome. We included this sentence in the discussion to be clear on this point:
"Current Hi-C data do not provide sufficient resolution to robustly identify sub-TADs or regulatory hubs within the Hoxb TAD in the developing mouse neural tube."

The development and emerging use of micro-C approaches, such as those used by Mike Levine's group to map regulatory hubs on a fine scale in early Drosophila embryos (Levo et al, Nature, 2022 & Batut et al, Science, 2022) hold promise for identifying and mapping sub- TAD/regulatory hub like domains. Efforts are being made to apply this approach to mammalian cell culture systems, but it has not been optimized for use in mammalian embryos and small amounts of tissue. We are very interested in using this approach to examine dynamics and properties in 3D organization of the Hoxb cluster in WT and RARE mutant embryos, as soon as it is feasible.

***Reviewer 2** (Advance Summary and Potential Significance to Field):*
In this manuscript from Afzal et al., the authors use single-cell, single-molecule approaches for interrogating the cis-regulatory logic of Hoxb genes in mouse embryos. It's an inspired choice for this type of analysis: the Hoxb locus has a complicated topology and is undoubtedly important for vertebrate development. The primary finding is that there is no evidence for co- transcriptional coupling of genes in the cluster. The authors then do a deep dive with mutagenesis of the various regulatory elements. Overall, I found the approach to be well-suited to the questions, but the narrative was hard to follow at times. There is a large body of work in the fly blastoderm on similar questions but little if any (to my knowledge) on vertebrates, let alone mouse. In that sense, this work is a technical advance. I think some revisions are required, but it seems like a strong fit for Development.

Reviewer 2 Comments for the Author:
Major comments:
1. a) There seems to be a substantial amount of background in the intronic signal which is usually very clean, necessitating a Deep Learning approach for transcription site segmentation. I don't think there is much to compare to when it comes to smFISH in mouse embryos, but the amount of fluorescence background diminishes my enthusiasm somewhat.

This is a good point which we would like to clarify. When we use intron only probes the signals are usually very clear and clean. However, Hox genes are relatively small and have single introns of variable size. This can make it challenging to design probes which only contain intronic sequences. Hence, in a few cases we needed to extend intron probes to include some sequences complementary to parts of exon to allow robust detection of nascent transcripts. This can increase background signal. We have modified Table S1 to include information on what regions are contained in each of the probes. To help clarify all this in the text we have added the following:

© 2023. Published by The Company of Biologists under the terms of the Creative Commons Attribution License (https://creativecommons.org/licenses/by/4.0/).

"To visualize nascent nuclear transcripts, we designed and validated probe sets spanning introns for genes of interest. In some cases, the probes only contained intron sequences, while in other cases the probes spanned intron and some exonic sequence due to constraints of gene size (Fig. 1B, Table S1)."
Also relevant to the issue of fluorescence background in embryos, we have been able to reliably detect single transcripts and nascents with low backgrounds for Hoxb1, Hoxa1, Cyp26, Nanog and other genes in ES cell culture models using smFISH (De Kumar et al Genome Research 2015 & De Kumar et al, PNAS 2017). However, in mouse tissue sections, while there clearly are single transcripts in the cytoplasm, we cannot robustly distinguish all of them from autofluorescence of the tissue. This is a common problem in fixed tissue sections. Hence, we focused on nascents. The DL learning pipeline was not required for separating nascent transcription sites from background, but to facilitate systematic collection and analysis of data from a large number of samples and diverse genetic backgrounds.
We have revised the text to say: "The cytoplasmic signal appears to represent single mature RNA transcripts but due to autofluorescence background present in the tissue section we were unable to robustly differentiate and count these signals. Hence, a caveat of our optimized smFISH protocol is that we could not quantify single mature transcripts, and measure total fluorescent intensity to infer transcriptional rates for genes (Fig. S1B)."

b) In addition, the co-localiztion distance of 1.5 um is rather large and raises eyebrows. Do any results change substantially with a cutoff of < 1 um? A false positive rate of 20% (taken from the Gapdh-Hoxb4 probe set) is an appreciable error.

We thank the reviewer for their comment about the spacing between co-localization sites of nascent transcription for probes from the same gene. We realize the way the data was originally presented made interpretation of the data very confusing. This resulted in a misleading impression on the levels of resolution of co-localized sites for different Hoxb4 probes and comparison to a Gapdh control. In the analysis presented in the original version of the paper, we use a nearest neighbor approach to find the closest spot for every individual spot in the data, without first thresholding away dim or small spots.

In the revised manuscript we re-analyzed and presented the data in a new way which should clear up the confusion. In this analysis, we identify all high confidence spots in the specific channel for each individual probe set (Hoxb4 intron, Hoxb4 exon and Gapdh). We then fit these spots to 2D gaussians to find their exact centers. Then we searched in a 3.9 x 3.9µm square around each of these spots for the presence of spots in channels for the other probes. This enabled us to calculate the distance between the centers of these high confidence spots. We used a histogram to plot the results for the distances between Hoxb4 intron vs Hoxb4 exon and Hoxb4 intron and Gapdh. These new plots are presented as Fig 2B and more clearly show what we were trying to convey in the original Fig.1D plot. As expected for probes against the same gene/transcript, we see that the majority of co-localized spots detected by Hoxb4 intron and exon probes are less that 500nm of each other, and the average distance between spots is 319nm. In contrast, the distance between Hoxb4 and Gapdh (different genes), is over 1.5µm. In addition, we quantified the fraction of spots that are less than 500nm apart for each Hoxb4 probe set and find that 64.5% of all Hoxb4 exon spots have a close by intron spot, while only 6.75% of Hoxb4 spots have a close by Gapdh spot. This colocalization data is now provided as a histogram in Fig. 2C.

To reflect this clarification and new analysis we have rewritten this section of text and replaced old Figs.1D,E with new Figs. 2B,C. The new text now reads:

"To further quantify the accuracy and robustness of our approach along with specificity of probes against nascent transcripts, we calculated the overlap of different Hoxb4 intron and exon probe sets (Fig. 2A). We compared distances between nascent transcripts observed from Hoxb4 intron versus exon probes against the distance between nascent transcripts observed for the Hoxb4 exon and Gapdh control probes. In tissue sections, we cannot demarcate cell and nuclear boundaries. Hence in each section we identified all high confidence spots in the specific channel for each individual probe set and fit these spots to 2D gaussians to find their exact centers. Then we searched in a 3.9 x 3.9µm square around each of these spots for the presence of spots in channels from the other probes and calculated the distance between the centers of these high confidence signals (Fig. 2B). As expected for probes against the same transcript, we see that the majority of

© 2023. Published by The Company of Biologists under the terms of the Creative Commons Attribution License (https://creativecommons.org/licenses/by/4.0/).

co-localized spots detected by Hoxb4 intron and exon probes are less than 500nm of each other, and the average distance between spots is 319nm. In contrast, the distance between spots of nascent transcription for the Hoxb4 and Gapdh genes is over 1.5µm (Fig. 2B). In addition, we quantified the fraction of spots that are co-localized less than 500nm apart and found that 64.5% of all Hoxb4 exon spots co-localize with a spot detected by the intron probe spot (Fig. 2C). The fraction of co-localized Hoxb4 intron and exon probes is lower than 100% which may be attributed to co-transcriptional splicing, as we capture spliced nascent transcripts with the exon probe, which would not be detected by the intron probe set.

Only 6.75% of Hoxb4 spots have a Gapdh spot less than 500 nm away, and the actual overlap of these probes within cells is likely to be even lower because some of the Gapdh-Hoxb4 spots might also include instances where nascent transcripts from each gene are at the periphery of two different cells, instead of being in the same cell."

2. a) There needs to be a more rigorous statistical treatment and comparison to some sort of null hypothesis for co-transcriptional bursting. The statistical treatment might consist of a Fisher's exact test.

We have conducted additional statistical analysis of the data in the original Fig. S7. Due to other changes to the text in the revised manuscript this modified figure is now Fig. S8. We compared observed co-localization values against those that would occur by random chance in WT, single and compound RARE mutants and calculated significant differences between them using a Wilcoxon signed-rank test. In the new Fig. S8 an asterisk (*) indicates cases where the p-value is less than 0.05, and we also include a new supplementary table (Table S4) which lists exact p- values for all of the samples.

b) The null hypothesis is more difficult: what is the random expectation for co-occurring transcription sites? Are the genes just independent (unaware of each other) or actually anti-correlated? The latter suggests an active regulatory process at the level of individual bursts. I don't think fig. S7 satisfies this requirement. The authors might take a look at a recent paper on coordinated transcription placed on biorxiv (https://doi.org/10.1101/2022.07.07.499202). At present, this conclusion is interesting but not quite substantiated: "This suggests the presence of layers of regulation and enhancer interactions governing patterns of nascent Hoxb expression may include some that restrict transcriptional co-activation."

This is an important point. We agree that the data in the original Fig. S7 is suggestive of active regulatory processes rather than them being independent of each other but does not substantiate it. Finding the frequency of co-localizations occurred less than random was part of what led us to measure distances between triple co-localized spots in mutant embryos in Fig. 6. Hence, the text referring to original Fig. S7 has been modified and we also moved it to the beginning of the final section of the results, which is now entitled: "Mutations in RAREs change the spatial relationships between promoters in the cluster". This section contains revised text describing the rationale for experiments in Fig. 6 and the interpretation of results, which this reviewer asked us to do in point 3 below. Original Fig. S7 is now Fig. S8.

We really appreciate the reviewer pointing out the interesting preprint by Bohrer and Larson, which we have now included in our references. This was indeed very relevant to our work, hence we have also incorporated some of its important points in the revisions to the text to help explain the rationale and interpretation of results for experiments in Fig. 6 (see below).

3. a) Figure 6 description probably needs to be completely re-written. The text is so spare as to be almost uninformative and seems like it was written in haste -- I read this part again and again and still didn't quite get it. One is not directly visualizing the enhancer, correct? These are only nascent RNAs made from transcription units. So what does this subsection title mean: "*Enhancer-promoter distances decrease in the RARE mutants*"? I suppose the conclusion is that deletion of an enhancer changes the spatial configuration of the locus (Fig. 6C), but that is a different conclusion.

We apologize for the lack of clarity and confusion related to text describing the rationale for experiments and the results in Fig. 6. We were not directly visualizing enhancers but the relative position of active promoters based on spots of nascent transcription. As the reviewer suggested we

© 2023. Published by The Company of Biologists under the terms of the Creative Commons Attribution License (https://creativecommons.org/licenses/by/4.0/).

have completely re-written this section and changed the title to: "Mutations in RAREs change the spatial relationships between promoters in the cluster". We also moved the data and analysis of the original Fig. S7 (now Fig. S8) to the beginning of this section as it helps to introduce the rationale for performing the experiments in Fig. 6. We have added a schematic to the top of Fig 6 (panel A) to illustrate how distances are measured between promoters. This shows how we envision the triangle plot (panel 6E) shows spatial relationships between promoters and how it is changed in RARE mutants.

The preprint by Bohrer and Larson presented several specific findings relevant to our analyses which we have incorporated into the revised text and discussion. One of their main conclusions is that genomic distance between genes is not predictive of whether they will occupy the same transcriptional hot spot or whether they will co burst. However, they find that the physical or spatial positioning of chromosomal loci is the driving factor in whether the genes/promoters are co-transcriptionally active and may occupy a common hub. They find that promoters that are positioned within a 1100nm physical space can influence interactions between each other when bursting. Furthermore, loci that are less than 1100nm apart when transcriptionally off, will move towards each other when they are transcriptionally active. Their results suggest that the positioning of genes in physical space influences the outcome of pairwise interactions: genes which are close to each other (MPD < 1100nm) move closer when bursting, and genes which are far from each other separate when bursting." This result fits nicely with our data as well. Our analyses which measured colocalized nascent RNA spots for three Hoxb co-bursting genes (Hoxb1, Hoxb4 & Hoxb9) indicated they were within 350nm of each other. This suggests they could be in a shared transcriptional hub despite differences in their genomic positions along the chromosome. Additionally, our Hoxb4 - Gapdh data shows that the distance between nascent spots for these genes is >1.5µm. Since these genes are located on different chromosomes, the distance between these genes suggests they are pulled into different transcriptional hot spots when active.

The study by Bohrer and Larson analyzed genes that are >1Mb apart on the chromosome. They note that for genes with "distances much less than 1Mb, therefore we could not determine how the correlation varies with increasing genomic distance for these values." We find it interesting that the entire Hox cluster is ~100kb, much smaller than any of the genomic distances in their study but the correlations appear to hold.

b) Is the plot in Fig. 6D showing that in the ENE-/- mutant, the whole locus is more condensed? i.e. area in the yellow triangle is smaller? This experiment seems like an immense amount of work, and maybe the conclusions are profound, but the interpretation is lacking for this referee.

The plot (now Fig. 6E) measures the distances between active promoters/nascent transcription of the three genes (Hoxb1, Hoxb4 and Hoxb9). The smaller size of the triangle indicates that the promoters are closer in the RARE mutants. Recent studies have shown that when a gene is not active it may change its relative spatial positioning. We were not able to incorporate DNA probes with the smFISH method to monitor spatial relationships of all genes in the cluster.
Based on changes in the positions of the three active promoters we show changes in distance for some genes but cannot for certain say that the whole cluster is condensed. As mentioned above, in response to point 3a, in the revised section describing Fig. 6, we present what we hope is a much more clear rationale for the experiments and the relevance of the interpretation of the data.

Minor comment:
1. I find it stylistically jarring to go back and forth between Figs. 1 and 2 out of order in the first part of the manuscript.

This is a good point also made by reviewer 3. Hence, we have generated new versions of Fig. 1 and Fig. 2 by rearranging panels from the original Figs. We have also replaced some panels with new analyses to clarify point 1b raised by this reviewer. These new versions of the Figs help the reader follow the logical flow of the text and analyses.

2. "A caveat of our optimized smFISH protocol is that we could not distinguish single transcripts." If you can't see single transcripts, is it really smFISH?

© 2023. Published by The Company of Biologists under the terms of the Creative Commons Attribution License (https://creativecommons.org/licenses/by/4.0/).

As we noted in response to point 1a of this reviewer (see above), we are able to detect single transcripts with our smFISH protocols in both ES cells and mouse embryos. However, the issue in mouse sections is that due to autofluorescence it is challenging to robustly differentiate and count the signals from single transcripts. To maintain robustness in our analyses we only focused on counting nascent which we can reliably do in tissue sections.

We have revised the text to say: "The cytoplasmic signal appears to represent single mature RNA transcripts but due to autofluorescence background present in the tissue section we were unable to robustly differentiate and count these signals. Hence, a caveat of our optimized smFISH protocol is that we could not quantify single mature transcripts and measure total fluorescent intensity to infer transcriptional rates for genes (Fig. S1B)."

3. Fig. 6 needs some sort of treatment of statistical significance.

This point is similar to point 8 raised by reviewer 3. There are no error bars on these distance plots because they are histograms. We have plotted the histograms as line plots rather than bars to avoid overcomplicating the figure. Multiple bar plot style histograms will require offsets or many subpanels to illustrate each histogram clearly. Rather than clutter the figure, we chose to plot the histograms as lines. We have changed the y axis title to "Occurrence frequency" in order to help clarify the nature of the plot.

***Reviewer 3** (Advance Summary and Potential Significance to Field):*
In this manuscript, Afzal and colleagues set out to decouple the mechanisms that regulate the expression of genes and non-coding RNAs within the Hoxb gene cluster. The authors focus on three shared retinoic acid response elements (RARE) enhancers, and develop an optimized smFISH protocol coupled with deep learning to measure nascent transcription for a subset of genes within the Hoxb cluster in mouse embryonic tissues. The authors report that nascent transcription tends to happen for a single Hoxb gene (of the 6 total elements analyzed) in individual cells at a given time. Single and a combination of mutations in different RARE enhancers affect both nascent transcription of individual genes and coordinated expression across genes and lnc within the cluster. The authors observe some structure in how different enhancer mutations affect specific patterns, suggesting that there are underlying regulatory mechanisms driving robustness in levels and patterns of Hoxb genes.

Overall, this is a well-designed and timely study, bringing in new experimental and computational techniques to mouse neural tube development. The manuscript is clearly motivated, the text is easy to follow, and the authors have made substantial efforts to perform appropriate controls as they bring in smFISH to this stage of mouse tissue. The results are generally supported by the data, and the authors are generally careful and cautious in their conclusions given their observations.

Reviewer 3 Comments for the Author:
I only have a few comments, which I have provided below for the authors to consider.

1. I found it a bit hard to go back and forth between Figure 1 and Figure 2 as they are not chronologically stated in the main text. The authors may consider starting with Figure 2 first, or at least combine early parts of Figure 1 with all of Figure 2 to avoid going back and forth between the two.

This is a good point also made by reviewer 2. Hence, we have generated new versions of Fig. 1 and Fig. 2 by rearranging panels from the original Figs. We have also replaced some panels with new analyses to clarify point 1b raised by reviewer 2. These new versions of the Figs help the reader follow the logical flow of the text and analyses.

2. I am not sure why the authors were not able to visualize single transcripts for any of the genes tested. While I commend the authors for clearly stating this observation, is this because the expression of mRNA is too high or barely detectable? I do not expect them to perform new experiments for this study as it is way beyond the scope of their work, but the authors may want to discuss in their discussion section if newer brighter clampFISH/HCR techniques could be used to address this in the future (e.g. Choi et al Development 2018, or Dardani et al Nature Methods 2022).

© 2023. Published by The Company of Biologists under the terms of the Creative Commons Attribution License (https://creativecommons.org/licenses/by/4.0/).

This point is related to major point 1a and minor point 2 raised by reviewer 2. As we noted in response to this reviewer (see above), we are able to detect single transcripts with our smFISH protocols in both ES cells (De Kumar et al Genome Research 2015 & De Kumar et al, PNAS 2017) and mouse embryos. However, the issue in mouse sections is that due to autofluorescence it is challenging to robustly differentiate and count the signals from single transcripts. This is a common problem in fixed tissue sections. To maintain robustness in our analyses we focused on counting nascents which we can reliably do in tissue sections. The DL learning pipeline was not required for separating nascent transcription sites from background, but to facilitate systematic collection and analysis of data from a large number of samples and diverse genetic backgrounds. The reason we chose not use clampFISH or HCR was to avoid an amplification step so we could detect nascent transcripts and quantify data across multiple wildtype and mutant samples.

We have revised the text to say: "The cytoplasmic signal appears to represent single mature RNA transcripts but due to autofluorescence background present in the tissue section we were unable to robustly differentiate and count these signals. Hence, a caveat of our optimized smFISH protocol is that we could not quantify single mature transcripts, and measure total fluorescent intensity to infer transcriptional rates for genes (Fig. S1B)."

3. Figure S3B is a bit confusing to me. Not sure if they need to compare sums across gene sets, and what that analysis reveals biologically. If the authors have a compelling reason for this analysis, I am curious to know.

These are not actually sums across gene sets. This data represents different embryos from multiple wildtype and mutant backgrounds analyzed for Hoxb4 expression using different probe sets containing Hoxb4. The key rationale for this plot is to illustrate the reproducibility of our method of probing and detection of nascent transcripts over different embryos and combinations of probes.

4. Figure 3F, the maximum dot is below 5. The authors should have the y-axis that is not too far from the maximum value. I understand they have it until 10 because they compare it to mutants in future figures, but for this figure, it is only appropriate to have the y-axis scaled such that there is not so much empty space.

This is a good point and we have revised Fig. 3F so that the y-axis now scales up to 4 rather than 10.

5. Figure S7: I do not see any statistical tests to confirm their observations. This is necessary for the authors to make the conclusion that co-localization is lower than by random chance.

This is related to point 2a raised by reviewer 2. We have conducted additional statistical analysis of the data in the original Fig. S7. Due to other changes to the text in the revised manuscript this modified figure is now Fig. S8. We compared observed co-localization values against those that would occur by random chance in WT, single and compound RARE mutants and calculated significant differences between them using a Wilcoxon signed-rank test. In the new Fig. S8 an asterisk (*) indicates cases where the p-value is less than 0.05, and we also include a new supplementary table (Table S3) which lists p-values for all of the samples.

6. Figure 4 and 5: the statistical significance with "*" and stars are not particularly clear. The authors should consider explaining it clearly in figure caption.

Due to constraints on total word count, we had provided the detailed explanation of the statistical analysis in the methods sections. The methods section entitled "Statistical analysis of data" explains that the asterisk (*) denotes significance in mean differences between nascent transcripts. There is also a description in that section for the variance that was calculated and shown in Fig. S7 (Fig. S8 in the original version).

7. Figure 5B: I like their plot, but I want to clarify to make sure I understand it the way the authors meant it: If each color is a different embryo, why are some colors less number than others (e.g. green for bimodal DE is only one dot. Does it mean that other green dots are hidden behind other dots? If so, the authors should add a small jitter to their plots to see all dots.)

© 2023. Published by The Company of Biologists under the terms of the Creative Commons Attribution License (https://creativecommons.org/licenses/by/4.0/).

There are two reasons for some dots not being visible. Yes, some dots are behind others. We have now included a small jitter and increased the width of the actual graphs, to better visualize the dots and avoid confusion between two adjacent samples. Another other reason for some samples being less represented than others was that during analysis, if a sample had blocks of high intensity background (from dust or other probe aggregations on the section), it distorted how DL could reliably count nascents. Hence, we excluded those samples from the final analysis. Exclusions were done with no regard to which sample the tissue section came from to avoid biasing the analysis.

8. Figure 6B: Why are there no error bars on the distance plots?

There are no error bars on these distance plots because they are histograms. We have plotted the histograms as line plots rather than bars to avoid overcomplicating the figure. Multiple bar plot style histograms will require offsets or many subpanels to illustrate each histogram clearly. Rather than clutter the figure, we chose to plot the histograms as lines. We have changed the y axis title to "frequency" in order to help clarify the nature of the plot.

Second decision letter

MS ID#: DEVELOP/2022/201259

MS TITLE: Shared retinoic acid responsive enhancers coordinately regulate nascent transcription of Hoxb coding and non-coding RNAs in the developing mouse neural tube

AUTHORS: Zainab Afzal, Jeffrey Lange, Christof Nolte, Sean McKinney, Christopher Wood, Ariel Paulson, Bony De Kumar, Jay Unruh, Brian D Slaughter, and Robb Krumlauf

I have now received all the referees reports on the above manuscript, and have reached a decision. The referees' comments are appended below, or you can access them online: please go to BenchPress and click on the 'Manuscripts with Decisions' queue in the Author Area.

The overall evaluation is extremely positive and we would like to publish a revised manuscript in Development. Before I can officially accept your manuscript I request that you address reviewer 2's point. Specifically, reviewer 2 requests that you clarify how you interpret "low numbers" of co-localization punctae. This would be a simple text addition for the point they indicate in your manuscript.

Please attend to all of the reviewers' comments in your revised manuscript and detail them in your point-by-point response. If you do not agree with any of their criticisms or suggestions explain clearly why this is so. If it would be helpful, you are welcome to contact us to discuss your revision in greater detail. Please send us a point-by-point response indicating your plans for addressing the refereeâ€™s comments, and we will look over this and provide further guidance.

Reviewer 2

Advance summary and potential significance to field

This work is a technical advance and sets a new bar for such measurements in vertebrate development. I think it should be published after a few small revisions.

Overall, I should add that I appreciate the extensive literature background, careful articulation of interpretations and rigorous data analysis. The work is quite scholarly in that regard.

© 2023. Published by The Company of Biologists under the terms of the Creative Commons Attribution License (https://creativecommons.org/licenses/by/4.0/).

Comments for the author

Wherever possible, I would like to see co-localization reported against some sort of null expectation. For example, they say “The low numbers of co-localized double or triple transcripts indicate that nascent transcription of Hoxb genes is not simultaneously activated in the same cell at the same time.” Low compared to what?
What is the expectation? This point is addressed later in the manuscript, but I think the reader is left hanging at this point and at a few other instances in the manuscript.

Reviewer 3

Advance summary and potential significance to field

The authors have addressed my questions. I am happy with the revised manuscript.

Comments for the author

The authors have addressed my questions. I am happy with the revised manuscript.

Second revision

Author response to reviewers' comments

Thank you for sending us the reviewers comments for the revised manuscript we submitted. I also appreciate your feedback that our plan to further revise the manuscript to address the last remaining point raised by reviewer 2 seemed acceptable. I have now incorporated those agreed changes into a second revised version of the manuscript which is uploaded onto the *Development Portal*.

Below I list the specific changes made to the manuscript to address the point of reviewer 2.

Comments of Reviewer 2:

Wherever possible, I would like to see co-localization reported against some sort of null expectation. For example, they say "*The low numbers of co-localized double or triple transcripts indicate that nascent transcription of Hoxb genes is not simultaneously activated in the same cell at the same time*." Low compared to what? What is the expectation? This point is addressed later in the manuscript, but I think the reader is left hanging at this point and at a few other instances in the manuscript.

Authors response:
This is a valid point. We feel there is a straight-forward way to resolve this issue by moving some relevant text to an earlier section and incorporating a few minor changes to the text to clarify what we mean by co-localizations and the basis for comparisons.

1. We explained and compared co-localization of nascent Hoxb transcription against the null expectation that correlations are a matter of random change in a late section of the first revision of the manuscript (Section: ***Mutations in RAREs change the spatial relationships between promoters in the Hoxb cluster*** page 19). Reviewer 2 notes that this was clear but feels it leaves the reader hanging without sufficient clarification in earlier parts of the paper.

We agree and have moved this explanation on frequency and meaning co-localizations to the earlier section (pages 14 & 15) in which we first describe the results on co-localized spots. This should remove any confusion and define what we mean by the occurrence of colocalizations from the very start. The text moved to this location (pages 14-15) now says:

© 2023. Published by The Company of Biologists under the terms of the Creative Commons Attribution License (https://creativecommons.org/licenses/by/4.0/).

"Analyses of the patterns of de novo transcriptional bursting activity from a single tail section (Fig. 3E) or from the average of multiple sections (Fig. 3G) clearly show that we predominately detect nascent transcription of a single gene in each cell, with a much lower frequency of simultaneous transcription of one or two other transcriptional units. For example, 72.2% percent of all sites of nascent transcription detected for Hoxb4 by the probe sets are found to occur as isolated single spots in a cell, with no evidence for nascent activity of the other Hoxb loci (Fig. 3E). In contrast, only 6.5% of Hoxb4 nascent spots are co-localized with Hoxb1, 18.1% with Hoxb9 and 3.2% with transcriptional bursts of both Hoxb1 and Hoxb9.
It was possible that the number of co-localized spots of nascent transcription we detected in individual cells was simply an outcome of random chance and the activities of each gene were independent of each other. Hence, we calculated the probability of random chance for co-localized transcripts based on the number of single nascent transcripts for each transcription unit. We found the actual observed co-localization trends were lower than what one would expect by random chance, suggesting there may be an active regulatory process modulating the levels of individual bursting activities (Fig. S5, Table S2)."

2. This rearrangement of text required modifying the order of some of the supplementary figures and tables, to reflect the new order in the text. This has been done.

3. *The confusing sentence the reviewer referred to: "The low numbers of co-localized double or triple transcripts indicate that nascent transcription of Hoxb genes is not simultaneously activated in the same cell at the same time." This sentence has been deleted as it is no longer required in light of the text which was moved.*

4. At the bottom of page 13 and top of page 14 one sentence was modified and another inserted to clarify what we mean by co-localized spots and how they relate to transcriptional activity.

<u>The old text said</u>: *"By imaging z-slices through a tail section, it is possible to visualize single and co-localized spots of nascent transcripts for the 3 coding genes, which we refer to as singles, doubles and triples (Fig. 3B,C)."*

The sentences now read: "By imaging z-slices through a tail section, we detected distinct single spots, where only one of the 3 loci is undergoing nascent transcription in a cell, and combinations of co-localized spots of nascent transcripts for these genes, which we refer to as singles, doubles and triples (Fig. 3B,C). The co-localized spots of nascent transcription for different combinations of Hoxb genes (doubles and triples) in an individual cell represent the occurrence of overlapping transcriptional bursting activities at these loci. Magnified insets illustrate examples of all of the combinations of overlapping nascent transcription observed in the tail section (Fig. 3D)."

We hope this satisfies the point by reviewer 2.

<u>Third decision letter</u>

MS ID#: DEVELOP/2022/201259

MS TITLE: Shared retinoic acid responsive enhancers coordinately regulate nascent transcription of Hoxb coding and non-coding RNAs in the developing mouse neural tube

AUTHORS: Zainab Afzal, Jeffrey Lange, Christof Nolte, Sean McKinney, Christopher Wood, Ariel Paulson, Bony De Kumar, Jay Unruh, Brian D Slaughter, and Robb Krumlauf
ARTICLE TYPE: Research Article

I am happy to tell you that your manuscript has been accepted for publication in Development, pending our standard ethics checks.

© 2023. Published by The Company of Biologists under the terms of the Creative Commons Attribution License (https://creativecommons.org/licenses/by/4.0/).
